# Record phenological responses to climate change in three sympatric penguin species

**DOI:** 10.1111/1365-2656.70201

**Published:** 2026-01-19

**Authors:** Ignacio Juarez Martinez, Alex Kacelnik, Fiona M. Jones, Jefferson T. Hinke, Michael J. Dunn, Andrea Raya Rey, Heather J. Lynch, Kate Owen, Tom Hart

**Affiliations:** ^1^ Department of Biology University of Oxford Oxford UK; ^2^ U.S. Antarctic Living Marine Resources Program, Southwest Fisheries Science Center, National Marine Fisheries Service, National Oceanic and Atmospheric Administration La Jolla California USA; ^3^ British Antarctic Survey Cambridge UK; ^4^ Centro Austral de Investigaciones Científicas, Consejo Nacional de Investigaciones Científicas y Técnicas Ushuaia Tierra del Fuego Argentina; ^5^ Instituto de Ciencias Polares, Ambiente y Recursos Naturales, Universidad Nacional de Tierra del Fuego Ushuaia Argentina; ^6^ Wildlife Conservation Society Representación Argentina Buenos Aires Argentina; ^7^ Department of Ecology & Evolution, Institute for Advanced Computational Science Stony Brook University Stony Brook New York USA; ^8^ Oxford Brookes University Oxford UK

**Keywords:** Antarctica, climate‐change, landscape ecology, monitoring, penguins, phenology, pygoscelids, time‐lapse camera

## Abstract

1. The timing of breeding is an important aspect of any species' realised niche, reflecting adaptations to synchronise with food supplies, dilute predation, avoid competition and exploit seasonal fluctuations in resources. Breeding phenology is typically studied either through long‐term monitoring of focal populations (limiting the strength of inferences about species‐wide traits and trends) or, when conducted at a landscape level, using remotely visible traits (restricting most studies to plants).
2. For the first time, this study demonstrates landscape‐scale measurement of vertebrate breeding phenology using a network of 77 time‐lapse cameras to monitor three sympatric penguin species across 37 colonies in the Antarctic Peninsula and Sub‐Antarctic islands.
3. Camera temperature loggers showed penguin colony locations are warming up four times faster (0.3°C/year) than the continental average (0.07°C/year), already the second fastest‐warming area in the world.
4. We analysed the start of the breeding season of Adélie, Chinstrap and Gentoo penguins at a sub‐continental scale between 2012 and 2022. The phenology of all three species advanced at record rates (10.2 ± 2, 10.4 ± 1.5 and 13 ± 4 days/decade, respectively).
5. Different demographic trends as well as intra‐ and inter‐species differences in response to environmental change suggest niche‐based response differences between species.
6. Phenological advances are causing niche separation to reduce. In this context, the Gentoo penguins' generalist and resident nature seems better suited to compete for space and resources than krill‐specialist Chinstraps and ice‐specialist Adélies.
7. Synthesis: A decade of observation of the three pygoscelid penguins shows they are advancing their settlement phenology at record speeds in relation to climate change across the Antarctic Peninsula. These changes are species‐dependent, reflecting different vulnerabilities and opportunities depending on their niche and life‐history traits. In the long term, the trend towards earlier settlement risks increasing inter‐species competition, causing trophic and temporal mismatch, and reshaping community assemblages.

## Graphical abstract

This paper is impressive because we managed to monitor extensively a really difficult place to reach and operate in. We deployed 77 cameras across Antarctica and the Sub Antarctic islands to monitor three different species of penguins. We found that they are the fastest advancing vertebrates with respect to their timing of breeding. We also found this is directly linked to environmental warming although each species is advancing in response to different environmental variables depending on their niches.

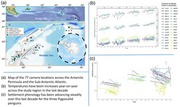

## Resumen

1. El momento escogido por una especie para reproducirse (fenología) es uno de los aspectos más importantes de su nicho ecológico por sus diversas funciones: maximizar el alimento disponible, minimizar la depredación, evitar la competencia y aprovechar las fluctuaciones estacionales de diferentes recursos. La fenología reproductiva se estudia normalmente mediante el seguimiento de unas pocas poblaciones a lo largo del tiempo, lo que limita la comprensión de tendencias a nivel de especie. Los pocos estudios de mayor rango geográfico utilizan variables visibles por satélite, restringiéndolos en su mayoría a plantas.
2. En este artículo proporcionamos por primera vez un estudio fenológico en tres especies simpátricas de vertebrados a nivel subcontinental usando una red de 77 cámaras. El estudio de las tres especies de pingüinos pigoscélidos (Adelia, Barbijo y Papúa) se realizó en 37 colonias de la península Antártica y las Islas Subantárticas entre 2012 y 2022.
3. La temperatura ambiente registrada por nuestras cámaras muestra un incremento acelerado de la temperatura en las colonias de pingüinos. En promedio +0,3 ºC/año cuatro veces más rápido que la media continental (0,07 ºC/año) a pesar de que ésta ya sea la segunda zona que sufre un calentamiento más acelerado a nivel planetario.
4. La fenología del comienzo de la temporada reproductiva (asentamiento) a nivel de especie avanzó a niveles récord: 10.2 ± 2, 10.4 ± 1.5 y 13 ± 4 días/década respectivamente para Adelia, Barbijo y Papúa.
5. La variabilidad inter e intraespecífica en las tasas de avance fenológico es compleja y parece estar relacionada con las diferentes adaptaciones de cada especie a su nicho ecológico.
6. Estos avances fenológicos están reduciendo la separación tradicional entre los nichos de las tres especies. En este contexto de mayor solapamiento temporal los Papúa (generalistas en su alimentación y residentes en invierno) podrían adquirir una ventaja competitiva con respecto a los Barbijo (especialistas en krill) o a los Adelia (especialistas en hielo marino), ambas migratorias en invierno.
7. Síntesis: Una década de observación de la fenología reproductiva de las tres especies del género *Pygoscelis* muestra niveles récord de avance en la fecha en la que anualmente escogen asentarse en la Península Antártica. Este avance está correlacionado con cambios ambientales en sus colonias y son característicos de cada especie, mostrando vulnerabilidades específicas durante el comienzo de la temporada reproductiva. De continuar esta tendencia, los pingüinos de la zona se exponen a varios riesgos: mayor competición interespecífica, desacople trófico y temporal respecto de sus presas y eventualmente un cambio en sus distribuciones.

## 1 INTRODUCTION

In seasonally breeding organisms, the timing of breeding (phenology) is a critical aspect of a species' realised niche that reflects adaptations to match ecological and environmental conditions for successful reproduction (Perrins, 2008; Visser & Both, 2005). Synchronising the period of maximal offspring growth to the timing of maximum availability (Perrins, 2008) or best quality (Rubenstein & Wikelski, 2003) of resources, however, may be difficult in the current period of rapid environmental change. In addition, the measurement of phenology is challenging at larger spatial scales.

The start of the reproductive season is a key phenological milestone that constrains the timing of subsequent life‐history events, and breeding as early as circumstances allow is often favoured (Ainley, 2002; Perrins, 2008). This is especially true for colonial birds, since early arrivals can occupy prime breeding spots (Bennett et al., 2022; Sergio et al., 2007) and enjoy higher reproductive success (Madsen et al., 2007; Verhulst & Nilsson, 2008), with more experienced birds breeding earlier and being more successful (Bennett et al., 2022; Daunt et al., 1999). Factors such as food availability at the breeding (Regehr & Rodway, 1999; Whelan et al., 2022) or wintering (Dobson et al., 2017) areas constrain breeding initiation, since starting the breeding season in good body condition is key for reproductive success (Vleck & Vleck, 2002).

Polar environments are characterised by extreme seasonality and phenology constrained by ‘hard limits’ to the season. For example, access to breeding or foraging areas (Madsen et al., 2007; Stenson & Hammill, 2014; Whelan et al., 2022) can be restricted due to snow or ice cover. To reconcile early arrival/breeding, optimal food availability and synchronicity (to minimise predation [Davis, 1982]), many species have evolved responses to biotic and abiotic cues. Temperature (Burnside et al., 2021; Samplonius et al., 2021), rainfall (Cayuela et al., 2014), plant phenology (McGrath et al., 2022), photoperiod (Dawson et al., 2001) or changes in food quality (Rubenstein & Wikelski, 2003) can all serve as proximate cues for the start of the breeding season. In migratory species, this extends to over‐wintering areas (Winkler et al., 2014).

Following external cues to optimise phenology during periods of rapid environmental change can cause changes in the timing of breeding. Many species are advancing their timing of breeding (Cole et al., 2021; Parmesan, 2006; Visser et al., 2012), others are delaying it (Barbraud & Weimerskirch, 2006; Dobson et al., 2017; Whelan et al., 2022) and some, including most seabirds (Keogan et al., 2018), are stable. These changes, in turn, can lead to a mismatch between predators and their prey (Parmesan, 2006; Samplonius et al., 2021; Thackeray et al., 2016; Visser et al., 2012), since different taxa follow different cues or respond differently to the same cues (Thackeray et al., 2010, 2016). Even if predators match their prey's shift (Matthysen et al., 2011; Parmesan, 2006; Visser et al., 2012), these changes might not be cost‐free, as breeding outside the usual window can incur costs like increased predation (Davis, 1982) or carry‐over effects (e.g. increased costs in the subsequent migration or breeding season; Fayet et al., 2016; Parmesan, 2006; Regehr & Rodway, 1999; Shoji et al., 2015).

In this study, we monitor the phenology of 37 colonies of three sympatric pygoscelid penguins that inhabit the Antarctic Peninsula (AP): the Adélie (*Pygoscelis adeliae*), Chinstrap (*P. antarcticus*) and Gentoo (*P. papua*). These species are key sentinels (Croxall et al., 2012) of the Southern Ocean due to their distribution throughout the Antarctic coastal regions and Sub‐Antarctic islands (Santora et al., 2020) and their role as key consumers of marine productivity alongside seals and whales (McCormack et al., 2021). Their strong fidelity to a colony also facilitates the consistent long‐term monitoring necessary to expose system change through time (Barbraud & Weimerskirch, 2006; Burr et al., 2016; Hinke et al., 2007).

Pygoscelid penguins nest on snow‐free ground, forming colonies that range in size between a dozen and hundreds of thousands of nests. In the AP, Adélies and Chinstraps are krill specialists, while Gentoos are generalists that switch to fish for large portions of their diet (Lynnes et al., 2004; Miller et al., 2009; Trivelpiece et al., 1987). In winter, Adélie and Chinstrap penguins migrate hundreds and thousands of kilometres away from their breeding colonies, while Gentoo penguins remain relatively local (Williams, 1995).

Despite their similarities and sympatry, the three species are currently experiencing diverging population trends. Adélie penguin populations have been decreasing throughout the AP, but are increasing in the cooler East Antarctica (Southwell et al., 2015). Chinstrap penguins are the most abundant of the genus, but in general decline throughout their range (Strycker et al., 2020). Gentoos, a more temperate species, are increasing in number and establishing new colonies throughout the AP (Clucas et al., 2015; Herman et al., 2020). However, population counts of such long‐lived species are not sensitive to real‐time variation in the environment because they integrate over longer time periods (Cerini et al., 2023; McDowall & Lynch, 2019). Demographic changes are trailing indicators that can only inform of disruptions after they have happened. Conversely, leading indicators like phenology are sensitive to real‐time conditions and are thus recommended to detect early disturbances to individual species (Cerini et al., 2023) and their environment (Croxall et al., 2012).

To overcome the challenging logistics of Antarctic work and obtain phenology estimates as an earlier metric of population disruption, we use an extensive network of 77 time‐lapse cameras. We describe the settlement phenology of the three species and their relationship with environmental changes over a period of 10 years across most of these species' breeding range in the AP and the Atlantic sub‐Antarctic islands. The AP was one of the fastest warming areas in the world in the late 20th century (Vaughan et al., 2003) and again during our study in the 2015–2020 period (Carrasco et al., 2021). Our aim was to quantify the magnitude of phenological shifts and to examine possible environmental drivers. Finally, we look at available data from the historical literature (Black, 2016) to compare the current situation to that of a pre‐warming AP.

## 2 MATERIALS AND METHODS

**FIGURE 1:**
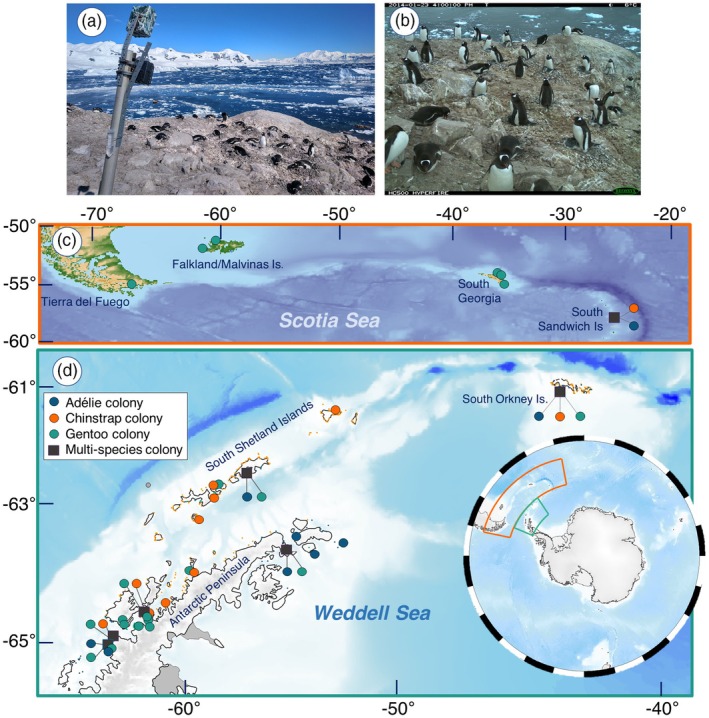
Study set‐up. (a) Example of monitoring cameras NEKOc and NEKOd at Neko Harbour, Andvord Bay. (b) Example image of NEKOc camera showing its field of view over several Gentoo nests. (c) Map of the Sub‐Antarctic study area, showing the geographic extent of the camera network across Tierra del Fuego, the Falklands‐Malvinas, South Georgia and the South Sandwich Islands. (d) Map of the Antarctic Study area including the Antarctic Peninsula, South Shetland Islands and the South Orkney Islands. Inset showing the location of the study system in the context of the Southern Ocean. Adélie colonies in this study are shown in blue, Chinstraps in orange and Gentoos in green.

In this study, we monitored 37 geographically separate penguin colonies between the 2011–2012 and 2021–2022 austral breeding seasons across the Antarctic Peninsula and the Atlantic Sub‐Antarctic Islands (Figure 1; See Appendix S1 for camera IDs, locations and abbreviations). Large or mixed species colonies (e.g. SIGN) required more than one camera, resulting in 77 time‐lapse camera monitoring locations (e.g. SIGNa, SIGNb, SIGNc). Each camera (Reconyx Hyperfire and Ultrafire; from Reconyx Inc., Holmen, WI, USA) was fixed to an aluminium scaffold pole secured using a tripod, a cairn, partial burial or a wire rock basket (Figure 1a). Cameras frame 10–25 nests in the foreground (Figure 1b; Southwell & Emmerson, 2015). Each unit was constructed during the austral summer (October to February), and serviced over subsequent summers, weather and ice conditions permitting. Cameras were programmed to record images and temperatures on the hour, 5–24 times a day.

Upon recovery, time‐lapse images were processed using custom R (version 4.0.5; R Core Team, 2025) scripts and Exiftool (exiftool.org; v12.8) to extract date, time and temperature metadata from each image. Images were referenced to a unique code including location, season and image number.

### 2.1 Settlement dates

To estimate settlement dates, we manually examined each camera‐ and season‐specific time series (i.e. camera‐seasons; *N* = 475) in a randomised order to avoid anchoring bias within colonies (Appendix S1). Of those, 277 camera‐seasons had enough quality images to reliably determine the settlement date. Discarded camera‐seasons did not have enough images due to battery depletion overwinter or because the camera lens was iced or otherwise obstructed.

In order to quantify the start of the breeding season we defined ‘Colony Settlement’ as the first date on which continuous daily presence of penguins at the nesting area in view began (far away parts of the colony in the background are ignored). We chose ‘Settlement’ over ‘Arrival’ (first recorded presence in the colony) partly because first records are by necessity extreme values and hence not representative of the population (Inouye et al., 2019). Moreover, Gentoos often have intermittent presence at their colonies throughout winter, rendering ‘first arrival’ inadequate. Clutch initiation dates were not considered as this paper focuses on breeding season start and the environmental processes that influence it. Also ‘Clutch Initiation’ estimation from cameras would have been too time‐consuming (Hinke et al., 2018) to carry out for the 277 camera‐seasons considered here.

### 2.2 Environmental variables

We used several environmental temperature variables to investigate the effect of warming on breeding behaviour. Two temperature variables were constructed using data from the camera's internal temperature logger from 11 AM to 1 PM, the period common to all cameras and years. The first variable, ‘median spring temperature’ (September to November), is a broad measure of the season's warmth at each colony. The second variable, ‘proportion of non‐freezing days in October’, is a proxy for ice/snow‐allowing conditions during settlement. October is the month in which, on average, the three species settle to breed.

Each camera contains a thermometer that records temperature as metadata on each image. We used camera SIGNa to validate temperature records relative to weather station standards by comparing 4 years of readings against a weather station located 1.5 km away in Signy Island (Polar Data Centre et al., 2021). On average, the camera recorded +1.8°C higher than the weather station (see Appendix S2 for temperature validation). We corrected all camera records of temperature data accordingly, assuming that the temperature sensors on all other cameras performed similarly.

Other environmental variables included were ‘Sea‐Ice concentration’ and ‘Net Primary Productivity’ using E.U. Copernicus Marine Service Information (https://doi.org/10.48670/moi‐00134 and https://doi.org/10.48670/moi‐00020, respectively). Five kilometres and 20 km buffer areas were drawn around each colony in order to extract the relevant data. See Appendix S3 for information on environmental variable construction.

### 2.3 Statistics

We modelled settlement using all three combinations of the four environmental variables plus latitude as fixed effects while keeping colony as a random effect to deal with pseudoreplication (there were more than one camera in some colonies, Appendix S3). Models were fitted using the *lmer* function (*lme4* package in R) and compared and checked for collinearity using the *performance* package. The final model was selected according to Akaike's Information Criterion (AIC); in case of a draw (ΔAIC <2), the model with the smallest AIC was kept. The *t*‐value is also presented for all fixed effects and marked red in their respective tables if |*t*| > 2 (the equivalent of a significant result in usual linear models). All other models (latitude, temperature rise) include colony as a random effect unless stated otherwise. Collinearity was low among all covariates in all models. Further details on model building and selection can be found in Appendix S3. Means and model outputs are expressed ± their standard error.

## 3 RESULTS

### 3.1 Decadal phenological shift

**FIGURE 2:**
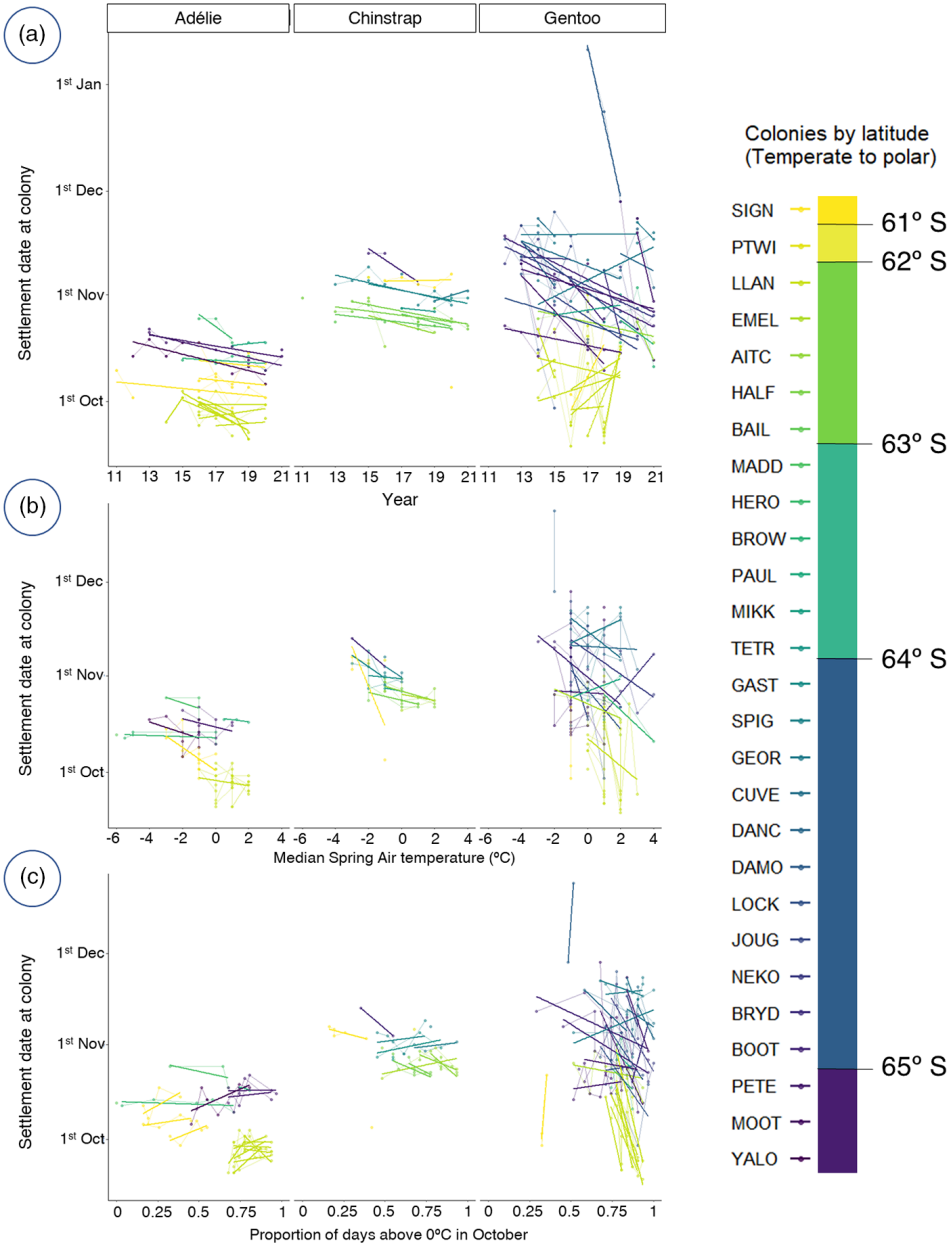
Colony settlement is advancing in Antarctic penguins in response to climate change. (a) Settlement date as estimated from time‐lapse cameras through the period of study. (b) Settlement date in relation to median spring air temperature, showing colony establishment occurs earlier in warmer years. (c) Settlement date in relation to the proportion of October days above zero. Settlement data for each camera is overlaid with their respective regression lines to ease interpretation and coloured according to their latitude from lower—more temperate—latitudes (brighter) to higher ‐more polar‐ latitudes (darker). Full details of all camera locations are provided in Appendix S1. For the equivalent plots on colonies in Sub‐Antarctic colonies, see Appendix S4.

Over the 10‐year study period, all three species shifted towards earlier colony settlement across all latitudes (Figure 2a). The rate and geographical pattern of this phenological advance differed between species.

#### 3.1.1 Adélie penguins

Adélies on the AP settled, on average, on the 15 October ±4.0 days, the earliest of the three species. During the study period, the settlement date advanced at a rate of −1.0 ± 0.2 days per year (*t*‐value = −4.9; details for all models in Appendix S3). This phenological shift was not uniform among Adélie colonies and varies among geographic regions (Figure 2a). The southernmost colonies (PETE and YALO) both showed a steady advance of the settlement date at a similar rate (purple; Figure 2a). All three colonies in Signy Island showed almost identical slopes despite their differences in settlement date (yellow; Figure 2a). Colonies in the Weddell Sea (BROW, HERO and PAUL in light blue) showed no definite trend (Figure 2a). The exception is LLAN, where some cameras recorded an advance while others were stable, indicating that intra‐colony variation in phenology can also be substantial. The northernmost Adélie colony (SSI) also advanced its settlement dates (see Appendix S4 for Sub‐Antarctic Islands figures).

#### 3.1.2 Chinstrap penguins

Chinstrap penguins settled on average on the 29 October ±6.4 days. Their settlement date advanced at a rate of −1.0 ± 0.1 days per year (*t*‐value = −6.6). All Chinstrap colonies in the AP displayed similar advances regardless of latitude (Figure 2a and Appendix S4). Chinstraps on Signy Island showed no specific trend.

#### 3.1.3 Gentoo penguins

Gentoo penguins on the AP settled on average on 1 November ±16.0 days. Their settlement dates spanned over a very wide ‘window’ of almost 6 weeks inside which they displayed great variability both within and among colonies (Figure 2a). Despite the greater variation in trends and rates, their phenology has been advancing at a greater rate than any of the other two species; this was true for the colonies on the AP (−1.3 ± 0.4 days/year; *t*‐value = −2.7) as well as for Sub‐Antarctic ones (−2.0 ± 1.2 days/year; *t*‐value = −1.7). Gentoo penguins in Sub‐Antarctic colonies displayed the earliest settlement dates, on average on the 4 October ±11.8 days (Appendix S4).

### 3.2 Latitudinal gradient

**FIGURE 3:**
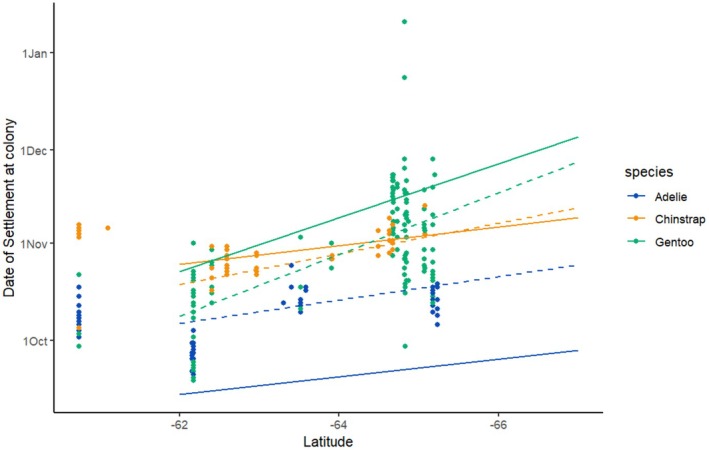
Settlement is influenced by latitude in the AP. Scatterplot with regression line showing the relationship between arrival dates and latitude for Adélie (blue), Chinstrap (orange) and Gentoo penguins (green) in colonies south of 62° S. Full lines indicate the output for the full model including environmental variables (see Table 1); dashed lines represent the output for a mixed model including only settlement in response to latitude and colony as a random effect. These models do not hold beyond 62° S as SIGN (60.7° S) and PTWI (61.1° S) settlement dates do not fit in with the models valid for the colonies containing the rest of the AP and south Shetland Island colonies.

Spatially, the phenology of all three species on the AP varied with latitude. Southern (more polar) colonies settled later than Northern (more temperate) ones (Figure 3). The relationship between latitude and colony settlement only applied to colonies south of 62° S. These trends did not hold for Antarctic colonies further north (SIGN and PTWI; Figure 3) nor for any Sub‐Antarctic colonies (Appendix S4).

### 3.3 Environmental factors

During the study period, we recorded widespread increases in the average midday temperatures in every colony outside the Weddell Sea (Appendix S3). The average temperature changes (°C/year ± S.E.) for the relevant months (once controlling for colony effect; Appendix S3) were 0.37 ± 0.05°C/year (August), 0.41 ± 0.08°C/year (September), 0.2 ± 0.05°C/year (October) and 0.44 ± 0.04°C/year (November). In all three species, phenological advance was related to at least one of our two temperature metrics (Figure 2b,c).

Adélie and Chinstrap penguin settlement dates were related to both ‘spring temperature’ and ‘freezing days’ (|*t*| > 2; Table 1). While settlement advanced with warmer spring air temperatures (Figure 2b, Table 1), a greater proportion of days above zero in October delayed settlement (Figure 2c, Table 1). Additionally, Adélie settlement was advanced by less ‘Winter Sea‐Ice’ while Chinstrap settlement advanced in relation to greater ‘Net Primary Productivity’ (Table 1).

**TABLE 1:** Model summary.

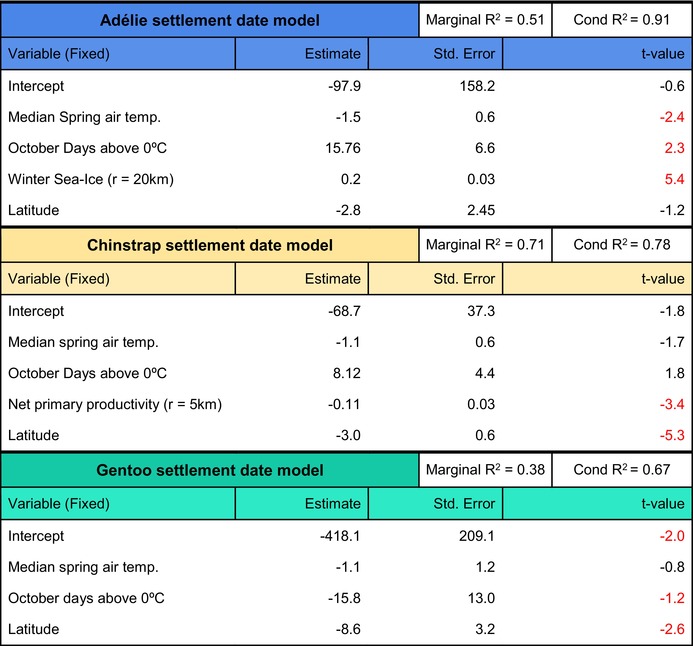

*Note*: The three models that best explain settlement for each species are shown below. These models were selected using AIC; Details on model building and selection in Appendix S3. *t*‐values are presented for all fixed effects and marked red if |*t*‐val| > 2 (the equivalent of a significant result in regular linear models).

### 3.4 Sub‐Antarctic colonies

In the Sub‐Antarctic (see Appendix S4), one Adélie and one Chinstrap colony at the South Sandwich Islands were monitored. Chinstraps advanced their settlement by 14.3 days per decade (*t* = −4.8), faster than their conspecifics in the AP. This is quite important since the South Sandwich Islands are home to the majority of the world's chinstrap population; Zavodovski Island is estimated to have 1.2 million individuals and the biggest penguin colony worldwide. Information for Adélies was very limited due to volcanic eruptions destroying their camera or sanding its lens in multiple seasons.

Gentoos showed a trend towards advancing settlement across multiple Atlantic Sub‐Antarctic colonies although this is not significant (Table S3.1). When modelled, latitude was the only variable to explain the differences in settlement between Sub‐Antarctic Gentoo colonies (Figures S4.1 and S4.2), rather than temperature (Tables S4.1 and S4.2). This is possibly due to milder temperatures in the Sub‐Antarctic not posing such hard limits (regarding snow/ice) to breeding as in the Antarctic.

### 3.5 Historical data

Comparing our settlement data with historical records for our study sites (see Black, 2016) showed differences between regions (see Appendix S6). In colonies at South Orkney, South Shetland and the Weddell Sea, ‘Return to Colony’ was recorded generally before or at the same time as our records for settlement. However, at all colonies on the Western Antarctic Peninsula our settlement estimates mostly occurred earlier than historical ‘Return to Colony’ data. Only Signy and Weddell Sea colonies overlapped with historical estimates of Arrival.

## 4 DISCUSSION

### 4.1 Record temperature increase

We conducted this study during a period of rapid warming across the Western Antarctic Peninsula (Appendices S2 and S3). This is a well‐established trend (Meredith et al., 2019) and there are concerns that this trend may negatively impact Antarctic biota (Kriegler et al., 2009). Spring air temperatures measured directly at our study colonies showed an unprecedented rate of increase throughout the AP over the last decade (Appendix S3: Figure S3.1 and Table S3.2). The rate of increase (0.34 ± 0.07 °C/year; Table S3.2) in the penguin colonies was several times greater than the last estimates of warming for Antarctica (+0.061 ± 0.034 °C /year; [Clem et al., 2020]) and specifically the Western Antarctic Peninsula (+0.36°C between 2015 and 2020; Carrasco et al., 2021). The pace of warming at colonies is not representative of the warming of the AP as a whole since our sampling locations are selected by penguins precisely for being ice‐free areas (Williams, 1995). The rapid on‐site warming trends imply that penguin colonies are some of the fastest‐warming locations on Earth.

### 4.2 Record phenological advance

Over the 10‐year study period, all three species advanced their settlement in breeding colonies, and this was related to warming in the region (Figure 2b; Table 1). Adélie and Chinstrap penguins showed significant advances in their settlement date at their Antarctic colonies at a rate of over 1 day per year (Table S3.1). Only colonies around the Weddell Sea showed no phenological advance: Signy for Chinstraps and Heroina, Madder Cliffs and Paulet for Adélies (Figure 2a). This is likely because these colonies have not experienced nearly as much warming (Figure S3.1) and suffered no sea‐ice loss (Figure S2.8) as also observed in seals by Dunn et al. (2025). Gentoo penguins have advanced their settlement at a rate of 13 days per decade (Table S3.1) with some colonies reaching 24 days per decade (Table S3.3). These trends were more variable in Gentoo penguins than for the other two species both between years and among colonies (Figure 2a). Phenological advance has happened in most individual colonies and also for each species' overall window for settlement (Figure 2a). The phenological variability among Gentoo penguins is consistent with previous studies on the phenology of incubation and hatching, that also demonstrate Gentoo's greater plasticity (Hinke et al., 2012; Lynch et al., 2012). These shifts were some of the fastest phenological changes ever observed in any animal (Romano et al., 2022). Relative to Cohen et al.'s (2018) global review of animal phenological responses to climate change, our data indicate Gentoos have undergone the fastest phenological shift on record for all bird species while Adélies and Chinstraps are respectively fourth and fifth. Relative to all vertebrates, these species would be first, fifth and sixth, respectively (Cohen et al., 2018).

It is difficult to establish how this shift compares to historical data. The available ‘Arrival’ data lacks standardisation (records were taken at different dates of the season and using various estimation methods by different people) and are often derived from opportunistic annotations in expedition logs (resulting in a disparity of locations and sampling effort). Thanks to our time‐lapse cameras, we know ‘Arrival’ is not suitable for the study of Gentoo penguins as they make multiple appearances overwinter and in the early season. Still, compared to Black's (2016) review on historical phenology we can see that our estimates of settlement happened earlier than the earliest recorded ranges of arrival dates for all three species (Appendix S6). As arrival necessarily happens before settlement, it is almost certain that settlement phenology has been advancing well beyond the oscillations in historical data. This was true for every colony except for those at the Weddell Sea (Appendix S6), which have not advanced their phenology as discussed above.

### 4.3 Environmental factors and species ecology

The phenological advance reported here was significantly correlated to a rapid increase in air temperatures for all three species (Figure 2b,c; Table 1) despite differences in their ecology. Adélie penguins showed a steady advance of settlement date through the decade with colonies displaying similar rates within but not across regions. Colonies within the Weddell Sea, South Shetland, South Orkney or Western AP all showed similar rates of advance (Figure 2a, Table 1). In all cases, the advance was related to warming (Table 1, Figures 4 and 5), but how it translated into regionally distinct phenological advance is a matter of speculation. Sea‐ice extent (found here related to settlement; Table 1) has been known to impact breeding start date for Adélies (Arrigo et al., 2002; Kooyman et al., 2007) but snowfall could also be distinct regionally.

Most Chinstrap colonies showed a very similar rate of advance in all colonies throughout their range (Figure 2a). Given their winter use of open maritime areas (Davis & Darby, 2012; Hinke et al., 2019), it is possible that the start of the breeding season was influenced by broad oceanic factors over local or regional events. Equivalent peninsula‐wide similarities in Chinstrap breeding phenology (e.g. egg lay and hatch dates) across their range have also been reported (Hinke et al., 2018).

Adélie and Chinstrap settlement dates were influenced by spring air temperature (Figure 2b; Table 1) and the percentage of days below freezing in October (Figure 2c; Table 1). Both species advanced their season in relation to an increase in overall spring temperatures (Figure 2b; Table 1); however, the loss of freezing days concurrently delayed their phenology (Figure 2c; Table 1). This shows a complex scenario since both variables are associated with warming yet significantly affect phenology shifts in opposite directions.

Previous studies estimated similar rates of phenology advance in relation to temperature increases to ours (Table 1) for clutch initiation dates in Adélies and Chinstraps (Hinke et al., 2012; Lynch et al., 2012). Rising temperatures might facilitate earlier breeding either through penguins using temperature as a proximate cue (Burnside et al., 2021; Winkler et al., 2014) or because of the melting of colony snow or sea‐ice that physically allows settlement (Madsen et al., 2007; Stenson & Hammill, 2014). These observations are consistent with the lack of advance in colonies around the Weddell Sea (Figure 2a) where we did not record a temperature (Figure S3.1) or sea‐ice (Figure S2.8) change during the study period.

Gentoos also showed a relationship between warming and earlier breeding; however, they advanced their phenology significantly in response to fewer freezing days only (Figure 2c, Table 1). It is difficult to identify which environmental process the loss of ‘freezing days’ captures since it advances Gentoo phenology while significantly delaying settlement in Adélies and Chinstraps (Figure 2c, Table 1). It is a variable related to environmental warming, yet it cannot be related to productivity or sea‐ice concentration near the colonies since these processes have their own non‐collinear variables in the model. ‘Freezing days’ must then be capturing some variability related to warming left unexplained by ‘Median Spring Temperatures’. We suggest that this variable captures differences in non‐breeding strategies that influence colony arrival. In winter, Adélie and Chinstrap penguins can migrate thousands of kilometres to occupy, respectively, marginal ice zones and open waters north of the ice edge, while Gentoos remain in the vicinity of their breeding colonies (Clausen & Pütz, 2003; Erdmann et al., 2011; Hinke et al., 2019). In increasingly warmer years (Figure S3.1), suitable habitats associated with the ice edge may be located farther from breeding locations. If so, the transit times back from wintering habitats to breeding colonies could be greater and would contribute to a delay in apparent settlement dates. This hypothesis remains to be tested in the field.

‘October Net Primary Productivity’ (NPP) increased around our study colonies (*r* = 5 Km) during the study period, especially since 2018 (Figure S2.6) and Chinstraps have been advancing their settlement in relation to it (Figure S2.7, Table 1). Increased NPP in the coastal AP over the last decade is thought to be a result of increased glacial melting, which favours phytoplankton blooms through water stratification and micronutrient enrichment above (Jack Pan et al., 2025; Krause et al., 2021). The differential response of Chinstraps to observed NPP increases could have been related to their differential foraging constraints as strict krill specialists (Lynnes et al., 2004; Rombolá et al., 2010; Trivelpiece et al., 2011). Gentoos, on the contrary, eat fish frequently (Miller et al., 2009, 2010) and Adélies can do so, but at the risk of increased foraging effort, impacts on breeding success and reduced juvenile recruitment (Fraser & Hofmann, 2003; Lynnes et al., 2004; McMahon et al., 2019). Food scarcity is the main and most common reason found to delay breeding onset in seabirds (Dobson et al., 2017; Regehr & Rodway, 1999; Whelan et al., 2022) including penguins (Davis & Darby, 2012; Trivelpiece et al., 2011; Vleck & Vleck, 2002), as individuals take longer to gain enough body condition to initiate breeding. We hypothesise that Chinstraps, which are limited to krill, are therefore more limited in their possibilities to feed during the early season. Consequently, they could have benefitted the most from increasing October NPP and this may allow them to settle earlier than their congeners. There are two non‐exclusive mechanisms by which this could have happened. Firstly, increased NPP and the environmental conditions that allowed it could have attracted more krill to the areas surrounding the study colonies. Krill are capable swimmers that can move to find prey (Tarling & Fielding, 2016), usually cued by low salinity (Tarling & Thorpe, 2014) and phytoplankton odour (Weissburg et al., 2019). Both cues have been observed in the coastal AP as a result of increased glacial melt during the study period (Jack Pan et al., 2025; Krause et al., 2021). Secondly, increased NPP may imply that krill, regardless of their numbers, were better fed and therefore more nutritious for penguins. Favourable environmental conditions can increase krill lipid quality in just a few weeks (Hellessey et al., 2020), something that can be detected from satellite‐derived sea surface temperature and chlorophyll‐a, precisely the components of our NPP estimates (Hellessey et al., 2020; Silk et al., 2016). Further research is needed to understand if these increases are enough to affect Chinstrap body condition in the early season and/or egg yolk composition, the latter seen as a limiting factor for embryo development in other penguins (Polito et al., 2012).

Winter sea‐ice cover decreased around (*r* = 20 km) our study colonies, especially since 2016 and especially in the southernmost colonies (YALO, PETE; Figure S2.8). We found Adélies to advance their settlement significantly in relation to sea‐ice loss (Figure S2.9, Table 1). During winter, Adélies occupy the coldest, more ice‐prone areas of these three species (Figure S2.8), possibly explaining why this species is the only one to see a response to sea‐ice loss. Understanding this mechanism would require further research, although sea‐ice has various known effects on their breeding cycle and performance (Emmerson & Southwell, 2008). Nearshore sea‐ice particularly has been shown to delay algal blooms (Arrigo et al., 2002), although these were not detected through our NPP estimates. Sea‐ice can also impact Adélie diet (Dugger et al., 2014; Wilson et al., 2016), as well as their breeding performance (Emmerson & Southwell, 2008). In extreme cases, there is anecdotal evidence of ice accumulation physically impeding access to the colony (Ainley, 2002).

All three species showed a significant trend in relation to latitude (Figure 3). Gentoos showed the greater effect of latitude on phenology; their southern colonies bred much later than their congeners, but the relationship was not as marked as in earlier studies (Black, 2016). This may reflect the different metrics used to establish the start of the season (arrival versus settlement date, as discussed above) or the more restricted geographical scope of our model (Figure 3).

The strong dependence on temperature‐related environmental variables suggests that fixed variables like daylight hours might not be the only environmental cues used to start the breeding season (Emmerson et al., 2011). Photoperiod serves a role in marking the time to return from migration (Dunn et al., 2011) and in stimulating hormone production in preparation for the season (Ainley, 2002). Such a signal can be a pre‐requisite of breeding initiation but could not explain the phenological advance seen here, since daylight hours are constant among years. Photoperiod‐induced hormone control might play a role in the future by serving as a hard limit on how early penguins can reproduce.

### 4.4 Increased competition

These three sympatric species have historically avoided competition through segregating their niches regarding: (i) breeding chronology, (ii) foraging behaviours and (iii) life history traits (Lynnes et al., 2004; Trivelpiece et al., 1987). However, phenological changes driven by sea‐ice loss and the overall increase in temperatures in the AP (Nicol, 2006; Walsh et al., 2020) could increase niche overlap and inter‐specific competition.

Observed phenological advances would affect their relative breeding chronology (Adélies–Chinstraps–Gentoos at most latitudes, Figure 3). Gentoo penguins are overall the last species to settle at their respective latitudes so their greater phenological advance (Table S3.1) weakens pygoscelids' traditionally staggered breeding chronology (understood to be a result of their respective sea‐ice tolerances; Trivelpiece et al., 1987). This reduces their temporal niche separation (Lishman, 1985) and will likely increase interspecific competition (Lynch et al., 2012; Trivelpiece et al., 1987).

This might also result in greater competition in the future for nest sites in mixed colonies (34% of our monitored AP colonies). Through our cameras, we observe how very early ‘Arrival’ of Gentoos (weeks and often months before any species settles) allowed them to occupy Adélie and Chinstrap nests for days. Gentoos were eventually displaced by the other two species upon their arrival (and immediate settlement) and before Gentoos' settlement. In the future, Gentoos may become more difficult to displace if they have settled or have active nests by the time another species arrives. This would give them an advantage in the competition for nesting spaces as seen in other cases of competition between migratory and non‐migratory birds (Ahola et al., 2007).

Regarding foraging behaviour, Gentoos' more flexible diet (McMahon et al., 2019; Miller et al., 2009, 2010) could become an advantage if environmental warming impacts krill recruitment (Fraser & Hofmann, 2003; Saba et al., 2014; Siegel & Loeb, 1995). Chinstraps would be greatly affected if krill stocks decline and Adélies could be impacted more subtly as discussed above (Lynnes et al., 2004). At the same time Gentoos' resident life history relies on open, ice‐free seas in winter (Korczak‐Abshire et al., 2021), something increasingly common in our study area (Figure S2.8). Both factors could have simultaneously facilitated the record phenological shift and expansion of Gentoos throughout the AP (Herman et al., 2020), and even in the Weddell Sea (Wethington et al., 2023) during the decade of study.

### 4.5 Winners and losers of climate change

Climate change allows generalist species to out‐compete specialists as the latter lose specialist habitat while generalists' higher phenotypic plasticity allows them to cope better with high environmental variance (Davey et al., 2012; Morley et al., 2019). Here, the increasingly subpolar conditions of the AP can likely favour generalists (Gentoos) at the expense of polar specialists: krill‐specialist Chinstraps and ice‐specialist Adélies. This contributes to a dynamic of ‘winners and losers of climate change’ among the three sympatric pygoscelid species (Clucas et al., 2015) supported by the observed population trends recorded by MAPPPD at the study colonies (Humphries et al., 2017; Appendix S5).

Over the last 10 years, Gentoo penguin numbers have increased steadily (Appendix S5) and the species has established new colonies throughout the AP, including into areas that were previously Adélie‐only territory (Wethington et al., 2023). In contrast, most of the Adélie and Chinstrap colonies in our study have declined in recent years (Appendix S5). Some of the few colonies that have not experienced a decline are those that have remained phenologically stable (Figure 2a), particularly the Adélie colonies in the Weddell Sea where warming (Figure S3.1) and loss of sea‐ice have not been significant (Kumar et al., 2021).

## 5 CONCLUSIONS

This study demonstrates a record advance in pygoscelid phenology as a consequence of unprecedented warming and environmental change on penguin colonies across the AP. This is a remarkable response to environmental change, especially as it happens beyond the range of historical oscillations. However, it is unknown whether this change is impacting the breeding success of the pygoscelid penguins or the shift is part of each species' adaptation to change. This is of concern since previous studies have demonstrated that phenological mismatch for Adélie penguins can arise from large inter‐annual environmental fluctuations (Youngflesh et al., 2017) and that phenological changes can affect breeding output in the three species (Hinke et al., 2012). Regardless of the impact of the phenological shift, it is unclear how much more phenological elasticity these species will be capable of displaying if temperatures keep rising at the current rate.

Finally, the use of a network of time‐lapse cameras has proven a cheap, autonomous, non‐invasive and reliable way to acquire standardised long‐term ecological data in one of the harshest environments on Earth. This ease of use has allowed us to apply a common methodology at a sub‐continental scale while allowing the results to be comparable between species and across colonies and years.

## AUTHOR CONTRIBUTIONS

Ignacio Juarez Martinez, Tom Hart, Alex Kacelnik, Heather J. Lynch and Fiona M. Jones conceived the ideas and designed the methodology; Tom Hart, Jefferson T. Hinke, Michael J. Dunn, Andrea Raya Rey, Ignacio Juarez Martinez, Fiona M. Jones, Heather J. Lynch, Kate Owen and Alex Kacelnik collected the data; Ignacio Juarez Martinez analysed the data and led the writing of the manuscript. All authors contributed critically to the drafts and gave final approval for publication.

## CONFLICT OF INTEREST STATEMENT

No authors report conflicts of interest of any sort.

## ETHICAL APPROVAL

Research conducted at Oxford University was reviewed by the Local Animal Welfare Board. Work at Signy Island and South Georgia was approved by the British Antarctic Survey Animal Welfare and Ethical Review Board, UK permits were issued by the Foreign, Commonwealth and Development Office. All research protocols for the LLAN colonies are approved by the Southwest Fisheries Science Center/Pacific Islands Fisheries Science Institutional Animal Care and Use Committee (# SWPI 2020‐01). All field research activities are permitted under the U.S. Antarctic Conservation Act (Permit #2017‐012). No animals were handled during the course of this study; environmental impact assessments were reviewed by the University of Oxford and the British Antarctic Survey. Permits for activities were provided by the UK Foreign & Commonwealth Office and the US National Science Foundation (ACA Permits 2012_WM_01, 2017_013, and 2022_002 for use of remote cameras in Llano Point/Copacabana Colonies).

## Supporting information


**Appendix S1.** Camera location and details.

**Appendix S2.** Environmental Variables.
**Table S2.1.** Table showing the slope and Intercept of the three linear models ran to assess the bias of our timelapse camera dataset (from cameras SIGNa to SIGNe) compared to a professionally measured air temperature from the nearest British Antarctic Survey (BAS) meteorological station.
**Figure S2.1.** Daily mean temperature values from our camera at Signy Island (SIGNa) are plotted against equivalent readings from the meteorological station at the British Antarctic Survey (BAS) station on Signy Island. Each datapoint is coloured according to the month they were measured in. A blue line showing the linear model fil is plotted in the foreground. A black line with 0 intercept and slope 1 representing perfect equivalence between both datasets is plotted in the background for reference.
**Figure S2.2.** Daily median temperature values from our camera at Signy Island (SIGNa) are plotted against equivalent readings from the meteorological station at the British Antarctic Survey (BAS) station on Signy Island. Each datapoint is coloured according to the month they were measured in. A blue line showing the linear model fil is plotted in the foreground. A black line with 0 intercept and slope 1 representing perfect equivalence between both datasets is plotted in the background for reference.
**Figure S2.3.** Daily maximum temperature values from our camera at Signy Island (SIGNa) are plotted against equivalent readings from the meteorological station at the British Antarctic Survey (BAS) station on Signy Island. Each datapoint is coloured according to the month they were measured in. A blue line showing the linear model fil is plotted in the foreground. A black line with 0 intercept and slope 1 representing an ideal equivalence between both datasets is plotted in the background for reference.
**Figure S2.4.** Monthly mean temperature values from our camera at Signy Island (SIGNa) are plotted against the equivalent mean from the meteorological station at the British Antarctic Survey (BAS) station on Signy Island. Each datapoint is coloured according to the month they were measured in. A blue line showing the linear model fit is plotted in the foreground with its corresponding confidence interval. A black line with 0 intercept and slope 1 representing an ideal equivalence between both datasets is plotted in the background for reference.
**Figure S2.5.** Monthly median temperature values from our camera at Signy Island (SIGNa) are plotted against the equivalent mean from the meteorological station at the British Antarctic Survey (BAS) station on Signy Island. Each datapoint is coloured according to the month they were measured in. A blue line showing the linear model fit is plotted in the foreground with its corresponding confidence interval. A black line with 0 intercept and slope 1 representing an ideal equivalence between both datasets is plotted in the background for reference.
**Figure S2.6.** Changes in Net primary Productivity 5 km around the study colonies in the Antarctic Peninsula over the study period.
**Figure S2.7.** Relationship between Net Primary Productivity and Settlement data for all years and colonies of study available. Indicative regression line shown for chinstraps as it was the only species for which there was a significant relationship (see Table 1 in the main article).
**Figure S2.8.** Changes in winter (Jul–Sep) Sea Ice Cover 20 km around the study colonies in the Antarctic Peninsula over the study period.
**Figure S2.9.** Relationship between winter Sea Ice cover and Settlement data for all years and colonies of study available. Indicative regression line shown for Adelies as it was the only species for which there was a significant relationship (see Table 1 in the main article).

**Appendix S3:** Model selection.
**Table S3.1.** Mixed models used to calculate rate of change rate in settlement date for each species over the last decade (Negative slope meaning shift to earlier dates). Models were performed using the *lmer* function within the *lme4* package in R. Settlement dates were tested against the continuous time variable year using colony. See Figure 2 in the main text for individual colony datapoints used in this model and their slopes.
**Table S3.2.** Mixed models of monthly temperature increase over the last decade used to calculate warming rate in the study area. Models were performed using the *lme* function within the *nlme* package in R. Average midday temperature data for each month was tested against the continuous time variable year using colony as a random effect. See Figure 3 for individual colony slopes and SM Figure 2 for the monthly temperature datapoints used in these models.
**Table S3.3.** Rates of advance per colony in settlement date for each species at each colony over the 2012‐2022 decade (Negative slope meaning shift to earlier dates). These were calculated using linear models (lm function within the lme4 package in R). Settlement dates were tested against the continuous time variable year. See Figure 2 in the main text for individual colony datapoints used in this model and their slopes.
**Figure S3.1.** Monthly average estimates of direct temperature measurements from in‐built camera thermometers by year. Data from each colony is joined by a line and coloured according to the latitude of the colony (Darker meaning more southern/polar).

**Appendix S4:** Sub‐Antarctic colonies.
**Figure S4.1.** Time series for the settlement date as estimated from Antarctic monitoring cameras for each species through the period of study. Settlement data for each camera is overlaid with their respective regression lines to ease interpretation and coloured according to their latitude from higher ‐more polar‐ latitudes (darker) to lower—more temperate—latitudes (brighter).
**Figure S4.3.** Time series for the settlement date as estimated from Gentoo Sub‐Antarctic monitoring cameras through the period of study. Settlement estimates for the different colonies are coloured according to the suggested species segregation north and south of the polar front as suggested by various authors. Coloured Sub‐Antarctic estimates are overlaid on top of Antarctic settlement estimates for the corresponding species.
**Table S4.1.** Model Selection for Scotia Sea Gentoos. Model selection was conducted including all variables of interest and dropping them off sequentially. Models are compared using AIC values although marginal and conditional R‐squared values are also shown to indicate the proportion of variance explained. This only includes Gentoo colonies north of latitude 60S.
**Table S4.2.** Model Summary. The model that best explains arrival of Gentoo penguins at their respective Scotia Sea colonies corresponds to the model including latitude as the fixed effects while controlling for colony as the random effect. For more on model selection, see Appendix S3.

**Appendix S5:** Population trends.
**Figure S5.1.** (Left) Total nest count for all Adélie colonies in this study present in the MAPPPD database. (Right) Same nest count data standardised to visualise trends.
**Figure S5.2.** (Left) Total nest count for all Chinstrap colonies in this study present in the MAPPPD database. (Right) Same nest count data standardised to visualise trends.
**Figure S5.3.** (Left) Total nest count for all Gentoo colonies in this study present in the MAPPPD database. (Right) Same nest count data standardised to visualise trends.

**Appendix S6.** Settlement data compared to historical arrival data.

## Data Availability

Data are available from the Zenodo Digital Repository https://doi.org/10.5281/zenodo.17642754 (Juarez Martinez et al., 2025).
