# Supplementary material for: Record phenological responses to climate change in three sympatric penguin species
Source: J Anim Ecol. 2026 Jan 19;95(3):455–69. doi: 10.1111/1365-2656.70201 (PMC12957737; doi:10.1111/1365-2656.70201)
Supplement: Supplementary file 1 — Appendix S1. Camera location and details. [file JANE-95-455-s001.docx]

## Appendix S1. Camera location and details

**Table 1:** Details on the camera codes used on this study (including location, season and number of images available) for each species. The camera codes used here are in compliance with those used by the MAPPPD project (www.penguinmap.com; Humphries et al., 2017). Abbreviations A.P (Antarctic Peninsula), F./M. (Falklands/Malvinas), S.G. (South Gerogia), S.Sh. (South Shetland Islands), W.S. (Weddell Sea)

| Species | Camera code | Colony name | Latitude | Longitude | Number of images | First season studied | Last season studied |
| --- | --- | --- | --- | --- | --- | --- | --- |
| Adélie | BROWb | Brown Bluff, A.P. | -63.521 | -56.889 | 13729 | s14-15 | s19-20 |
|  | HEROa | Heroina Island, W.S. | -63.4 | -54.6 | 6956 | s15-16 | s17-18 |
|  | LLANc | Copacabana/Llano Point,  South Shetland Islands | -62.1763 | -58.4485 | 17111 | s14-15 | s19-20 |
|  | LLANd |  | -62.1763 | -58.4485 | 8551 | s17-18 | s19-20 |
|  | LLANe |  | -62.1768 | -58.4489 | 18887 | s13-14 | s18-19 |
|  | LLANf |  | -62.1768 | -58.4489 | 11617 | s13-14 | s19-20 |
|  | LLANm |  | -62.1768 | -58.4489 | 8547 | s16-17 | s19-20 |
|  | LLANp |  | -62.1759 | -58.4461 | 17185 | s14-15 | s18-19 |
|  | LLANq |  | -62.1749 | -58.4457 | 19617 | s14-15 | s19-20 |
|  | MADDa | Madder Cliffs, W.S. | -63.3 | -56.48 | 4218 | s16-17 | s18-19 |
|  | PAULa | Paulet Island, W.S. | -63.58 | -55.78 | 23676 | s17-18 | s20-21 |
|  | PETEd | Petermann Island, A.P. | -65.172 | -64.136 | 36044 | s12-13 | s18-19 |
|  | SIGNb | Signy Island, South Orkney | -60.737 | -45.657 | 8781 | s15-16 | s20-21 |
|  | SSISc | Saunders, S. Sandwich Is. | -57.805 | -26.42 | 878 | s14-15 | s14-15 |
|  | YALOa | Yalour Islands, A.P. | -65.235 | -64.158 | 28082 | s11-12 | S21-22 |
| Adélie & Chinstrap | SIGNa | Signy Island, South Orkney | -60.737 | -45.657 | 9731 | s10-11 | s20-21 |
| Adélie & Gentoo | PETEe | Petermann Island, A.P. | -65.172 | -64.136 | 27668 | s12-13 | s17-18 |
| Chinstrap | AITCd | Aitcho Barrientos Is., S.Sh. | -62.406 | -59.74 | 20402 | s14-15 | s19-20 |
|  | BAILa | Bailey Head, S.Sh. | -62.964 | -60.504 | 18721 | s12-13 | s20-21 |
|  | BAILb | Bailey Head, S.Sh. | -62.964 | -60.504 | 12005 | s16-17 | s20-21 |
|  | EMELa | Emeline Island, S.Sh. | -62.3918 | -59.7928 | 172 | s19-20 | s19-20 |
|  | GASTa | Gaston Island, A.P. | -64.4877 | -61.8358 | 10523 | s14-15 | s19-20 |
|  | GEORd | Georges Point, A.P. | -64.67 | -62.621 | 18586 | s15-16 | s17-18 |
|  | GEORe | Georges Point, A.P. | -64.669 | -62.6698 | 1842 | s18-19 | s19-20 |
|  | HALFa | Half Moon Island, S.Sh. | -62.595 | -59.899 | 2135 | s10-11 | s11-12 |
|  | HALFb | Half Moon Island, S.Sh. | -62.595 | -59.899 | 16861 | s12-13 | s20-21 |
|  | HALFc | Half Moon Island, S.Sh. | -62.596 | -59.898 | 30794 | s12-13 | s19-20 |
|  | PTWIa | Point Wild, S.Sh. | -61.099 | -54.859 | 2850 | s13-14 | s14-15 |
|  | PTWIb | Point Wild, S.Sh. | -61.099 | -54.859 | 4277 | s14-15 | s15-16 |
|  | SIGNc | Signy Island, South Orkney | -60.737 | -45.657 | 7858 | s15-16 | s20-21 |
|  | SPIGa | Orne Harbour, A.P. | -64.631 | -62.555 | 27667 | s12-13 | s19-20 |
|  | SSISa | Saunders, S. Sandwich Is. | -57.808 | -26.398 | 8729 | s13-14 | s15-16 |
|  | SSISb | Saunders, S. Sandwich Is. | -57.808 | -26.398 | 7182 | s13-14 | s16-17 |
|  | TETRa | Tetrad Islands, A.P. | -63.9166 | -60.733 | 5976 | s16-17 | s19-20 |
| Chinstrap & Gentoo | BOOTc | Booth Island, A.P. | -65.0665 | -64.0235 | 15799 | s14-15 | s18-19 |
| Gentoo | AITCb | Aitcho Barrientos Is., S.Sh. | -62.4057 | -59.7451 | 9968 | s13-14 | s18-19 |
|  | AITCc | Aitcho Barrientos Is., S.Sh. | -62.4078 | -59.747 | 23328 | s13-14 | s19-20 |
|  | BEAVa | Beaver Island, F./M. | -51.8389 | -61.2777 | 14404 | s14-15 | s16-17 |
|  | BLEAb | Bleaker Island, F./M. | -52.2 | -58.85 | 17240 | s17-18 | s17-18 |
|  | BOOTa | Booth Island, A.P. | -65.067 | -64.037 | 18189 | s11-12 | s20-21 |
|  | BOOTb | Booth Island, A.P. | -65.067 | -64.022 | 23824 | s12-13 | s19-20 |
|  | BROWa | Brown Bluff, W.S. | -63.522 | -56.889 | 2973 | s10-11 | s14-15 |
|  | BROWc | Brown Bluff, W.S. | -63.52 | -56.893 | 4419 | s14-15 | s18-19 |
|  | BRYDa | Bryde Island East, A.P. | -64.889 | -62.927 | 7379 | s15-16 | s19-20 |
|  | COOPc | Cooper Bay, S.G. | -54.783 | -35.802 | 12073 | s14-15 | s19-20 |
|  | CUVEa | Cuverville, A.P. | -64.682 | -62.621 | 34098 | s11-12 | s20-21 |
|  | CUVEb | Cuverville, A.P. | -64.682 | -62.621 | 14135 | s12-13 | s18-19 |
|  | DAMOa | Damoy Point, A.P. | -64.8175 | -63.519 | 12960 | s13-14 | s19-20 |
|  | DANCa | Danco Island, A.P. | -64.728 | -62.5965 | 11666 | s11-12 | s20-21 |
|  | DANCb | Danco Island, A.P. | -64.728 | -62.5965 | 14576 | s12-13 | s15-16 |
|  | GEORa | Georges Point, A.P. | -64.67 | -62.669 | 27085 | s12-13 | s19-20 |
|  | GEORb | Georges Point, A.P. | -64.669 | -62.6698 | 17671 | s13-14 | s19-20 |
|  | GODTa | Godthul, S.G. | -54.2 | -36.3 | 11886 | s15-16 | s18-19 |
|  | GODTb | Godthul, S.G. | -54.2 | -36.3 | 98382 | s15-16 | s15-16 |
|  | JOUGa | Jougla Point, A.P. | -64.826 | -63.493 | 19345 | s14-15 | s19-20 |
|  | LLANa | Copacabana/Llano Point,  South Shetland Islands | -62.1771 | -58.4461 | 23764 | s13-14 | s19-20 |
|  | LLANb |  | -62.1771 | -58.4461 | 22589 | s13-14 | s19-20 |
|  | LLANg |  | -62.1751 | -58.4483 | 21503 | s14-15 | s19-20 |
|  | LLANh |  | -62.1751 | -58.4483 | 8524 | s17-18 | s19-20 |
|  | LLANi |  | -62.1742 | -58.4471 | 23742 | s13-14 | s19-20 |
|  | LLANj |  | -62.1742 | -58.4471 | 22949 | s13-14 | s19-20 |
|  | LOCKa | Port Lockroy, A.P. | -64.8268 | -63.4929 | 20681 | s11-12 | s19-20 |
|  | LOCKb | Port Lockroy, A.P. | -64.825 | -63.492 | 39286 | s12-13 | s19-20 |
|  | MAIVa | Maiviken, S.G. | -54.249 | -36.49 | 207 | s14-15 | s14-15 |
|  | MAIVb | Maiviken, S.G. | -54.249 | -36.49 | 4675 | s12-13 | s13-14 |
|  | MAIVc | Maiviken, S.G. | -54.249 | -36.49 | 37489 | s12-13 | s19-20 |
|  | MARTf | Isla Martillo, Patagonia | -54.9067 | -67.3872 | 57589 | s12-13 | s17-18 |
|  | MIKKa | Mikkelsen Harbour | -63.902 | -60.79 | 12010 | s14-15 | s19-20 |
|  | MOOTa | Moot Point, A.P. | -65.204 | -64.075 | 3674 | s14-15 | s18-19 |
|  | NEKOa | Neko Harbour, A.P. | -64.844 | -62.529 | 23161 | s11-12 | s19-20 |
|  | NEKOb | Neko Harbour, A.P. | -64.8448 | -62.5296 | 6449 | s11-12 | s12-13 |
|  | NEKOc | Neko Harbour, A.P. | -64.843 | -62.528 | 17573 | s12-13 | s19-20 |
|  | OCEAa | Ocean Harbour, S.G. | -54.3414 | -36.2474 | 6783 | s14-15 | s15-16 |
|  | PETEa | Petermann Island, A.P. | -65.172 | -64.137 | 29303 | s11-12 | s20-21 |
|  | PETEc | Petermann Island, A.P. | -65.171 | -64.137 | 25926 | s12-13 | s18-19 |
|  | SANDa | Sandebugten, S.G. | -54.324 | -36.34 | 6785 | s14-15 | s15-16 |
|  | SAUNa | Saunders, the Neck, F./M. | -51.306 | -60.241 | 14181 | s12-13 | s19-20 |

References:

Humphries, G. R. W., Naveen, R., Schwaller, M., Che-Castaldo, C., McDowall, P., Schrimpf, M., & Lynch, H. J. (2017). Mapping Application for Penguin Populations and Projected Dynamics (MAPPPD): Data and tools for dynamic management and decision support. *Polar Record*, *53*(2), 160–166. https://doi.org/10.1017/S0032247417000055
