# Supplementary material for: Record phenological responses to climate change in three sympatric penguin species
Source: J Anim Ecol. 2026 Jan 19;95(3):455–69. doi: 10.1111/1365-2656.70201 (PMC12957737; doi:10.1111/1365-2656.70201)
Supplement: Supplementary file 2 — Appendix S2. Environmental Variables. Table S2.1. Table showing the slope and Intercept of the three linear models ran to assess the bias of our timelapse camera dataset (from cameras SIGNa to SIGNe) compared to a professionally measured air temperature from the nearest British Antarctic Survey (BAS) meteorological station. Figure S2.1. Daily mean temperature values from our camera at Signy Island (SIGNa) are plotted against equivalent readings from the meteorological station at the British Antarctic Survey (BAS) station on Signy Island. Each datapoint is coloured according to the month they were measured in. A blue line showing the linear model fil is plotted in the foreground. A black line with 0 intercept and slope 1 representing perfect equivalence between both datasets is plotted in the background for reference. Figure S2.2. Daily median temperature values from our camera at Signy Island (SIGNa) are plotted against equivalent readings from the meteorological station at the British Antarctic Survey (BAS) station on Signy Island. Each datapoint is coloured according to the month they were measured in. A blue line showing the linear model fil is plotted in the foreground. A black line with 0 intercept and slope 1 representing perfect equivalence between both datasets is plotted in the background for reference. Figure S2.3. Daily maximum temperature values from our camera at Signy Island (SIGNa) are plotted against equivalent readings from the meteorological station at the British Antarctic Survey (BAS) station on Signy Island. Each datapoint is coloured according to the month they were measured in. A blue line showing the linear model fil is plotted in the foreground. A black line with 0 intercept and slope 1 representing an ideal equivalence between both datasets is plotted in the background for reference. Figure S2.4. Monthly mean temperature values from our camera at Signy Island (SIGNa) are plotted against the equivalent mean from the meteorological station at [file JANE-95-455-s004.docx]

## Appendix S2: Environmental Variables

1. **Temperature validation**

Before using the temperature data from the in-built camera temperature logger we conducted several analyses to validate the accuracy and bias of our dataset against the temperature readings of a nearby meteorological station. We ground-truthed data from timelapse (TL) cameras SIGNa to SIGNe located on Signy Island (Appendix S1) against their corresponding measurements from the meteorological station located at the British Antarctic Survey (BAS) research base on Signy Island (Polar Data Centre, U. K et al., 2021). It is worth noting that the camera readings correspond only to the 11AM 12AM and 1PM measurements, since those are the only measurements common to all cameras and years.

First we tested our dataset to determine if it is unbiased. To do this we derived several daily parameters (Mean, median, maximum temperature) in both datasets and tested them against each other. We plotted these daily values for a visual interpretation (plots S2.1, S2.2 and S2.3) as well as running linear models using the *lm* function in R (Table S2.1). In an ideal scenario of no bias across the temperature range the slope of the model should be 1 and the intercept zero.

Observing plots S2.1, S2.2 and S2.3 demonstrates that the TL camera temperature estimates are biased across the range of temperatures. Mean and median temperature data derived from the camera is 1.8 and 1.6 degrees warmer than that of BAS logs (plots S2.1 and S2.2; Table S2.1). This is partly due to the TL dataset logging only midday temperatures rather than to a consistent bias across the temperature range since when we compare maximum temperatures the difference between datasets is reduced to 1 degree (plot S2.3; Table S2.1). That means the cameras record temperature that are on average 1 degree warmer than the BAS readings. This bias is consistent throughout the temperature range. This can be observed in S2.1, S2.2 and S2.3 and is confirmed by the slopes of the linear models; all of them very close to 1 (Table S2.1).

**Table S2.1:** Table showing the slope and Intercept of the three linear models ran to assess the bias of our timelapse camera dataset (from cameras SIGNa to SIGNe) compared to a professionally measured air temperature from the nearest British Antarctic Survey (BAS) meteorological station.

| Plot | Dependent variable (y) | Independent variable (x) | Slope | Intercept |
| --- | --- | --- | --- | --- |
| S2.1 | Daily Mean temperature SIGN | Daily Mean temperature BAS | 0.999 | 1.807 |
| S2.2 | Daily Median temperature SIGN | Daily Median temperature BAS | 0.979 | 1.621 |
| S2.3 | Daily Maximum temperature SIGN | Daily Maximum temperature BAS | 1.009 | 1.050 |


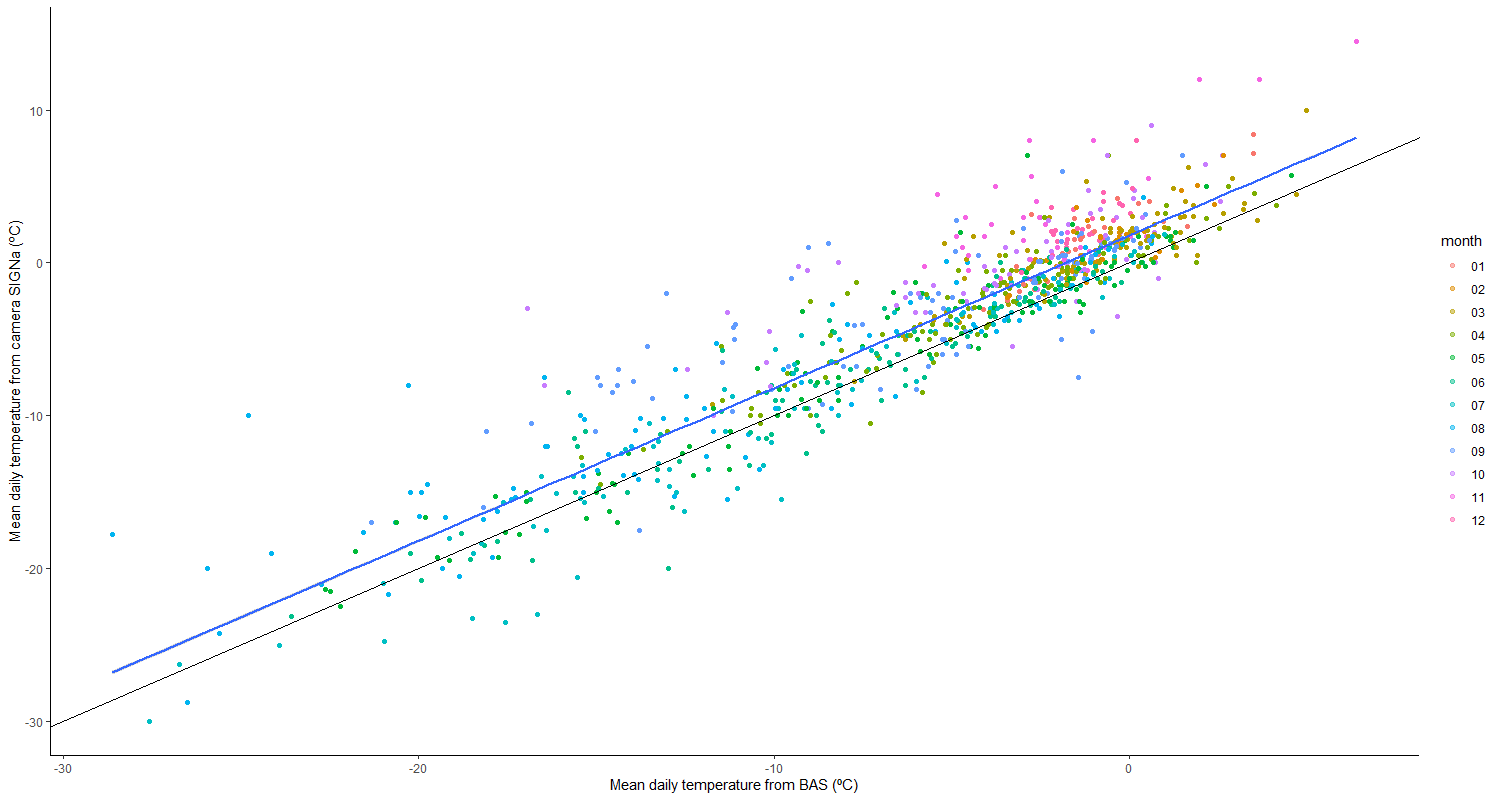


**Figure S2.1:** Daily mean temperature values from our camera at Signy Island (SIGNa) are plotted against equivalent readings from the meteorological station at the British Antarctic Survey (BAS) station on Signy Island. Each datapoint is coloured according to the month they were measured in. A blue line showing the linear model fil is plotted in the foreground. A black line with 0 intercept and slope 1 representing perfect equivalence between both datasets is plotted in the background for reference.


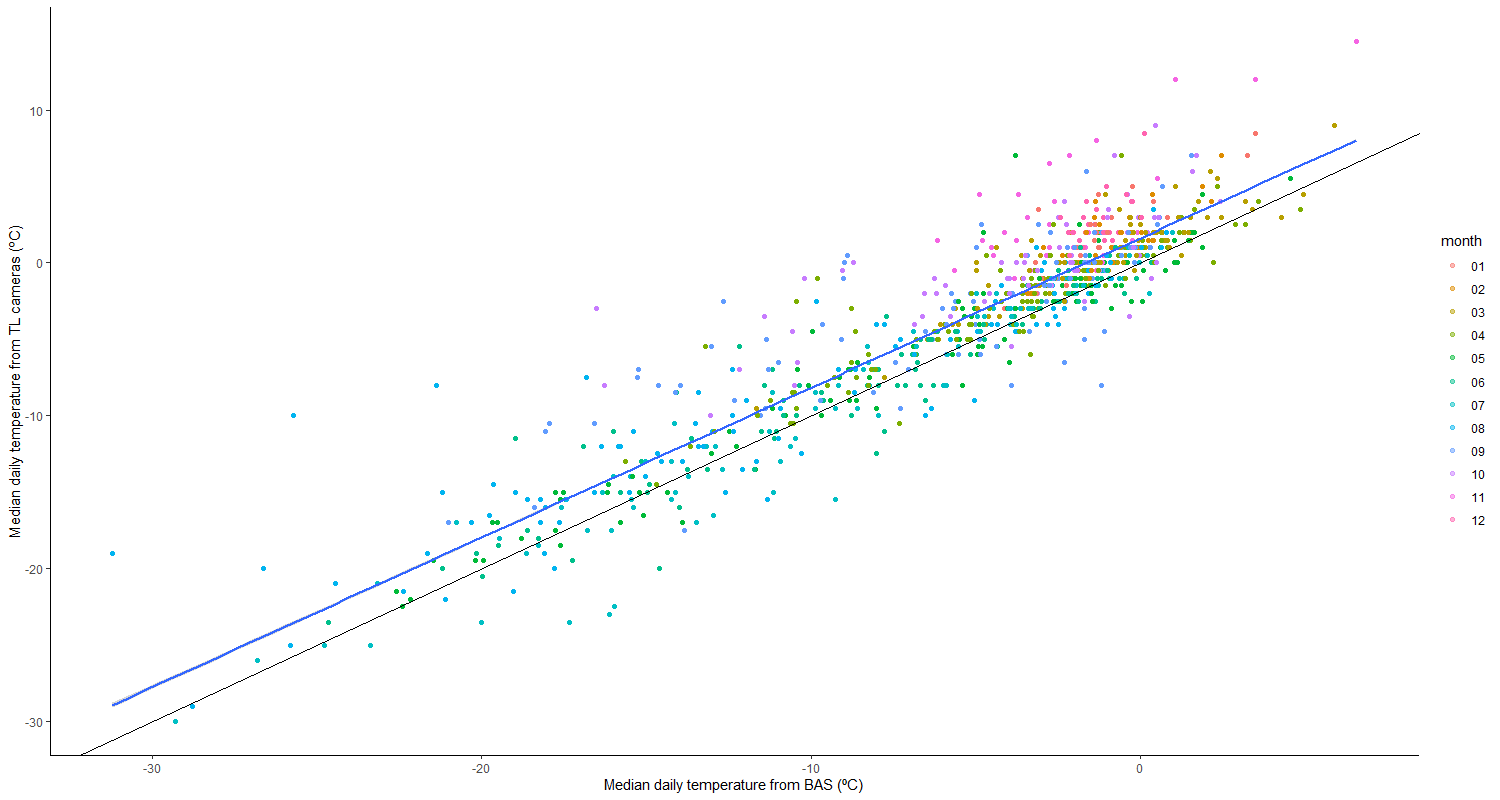


**Figure S2.2:** Daily median temperature values from our camera at Signy Island (SIGNa) are plotted against equivalent readings from the meteorological station at the British Antarctic Survey (BAS) station on Signy Island. Each datapoint is coloured according to the month they were measured in. A blue line showing the linear model fil is plotted in the foreground. A black line with 0 intercept and slope 1 representing perfect equivalence between both datasets is plotted in the background for reference.


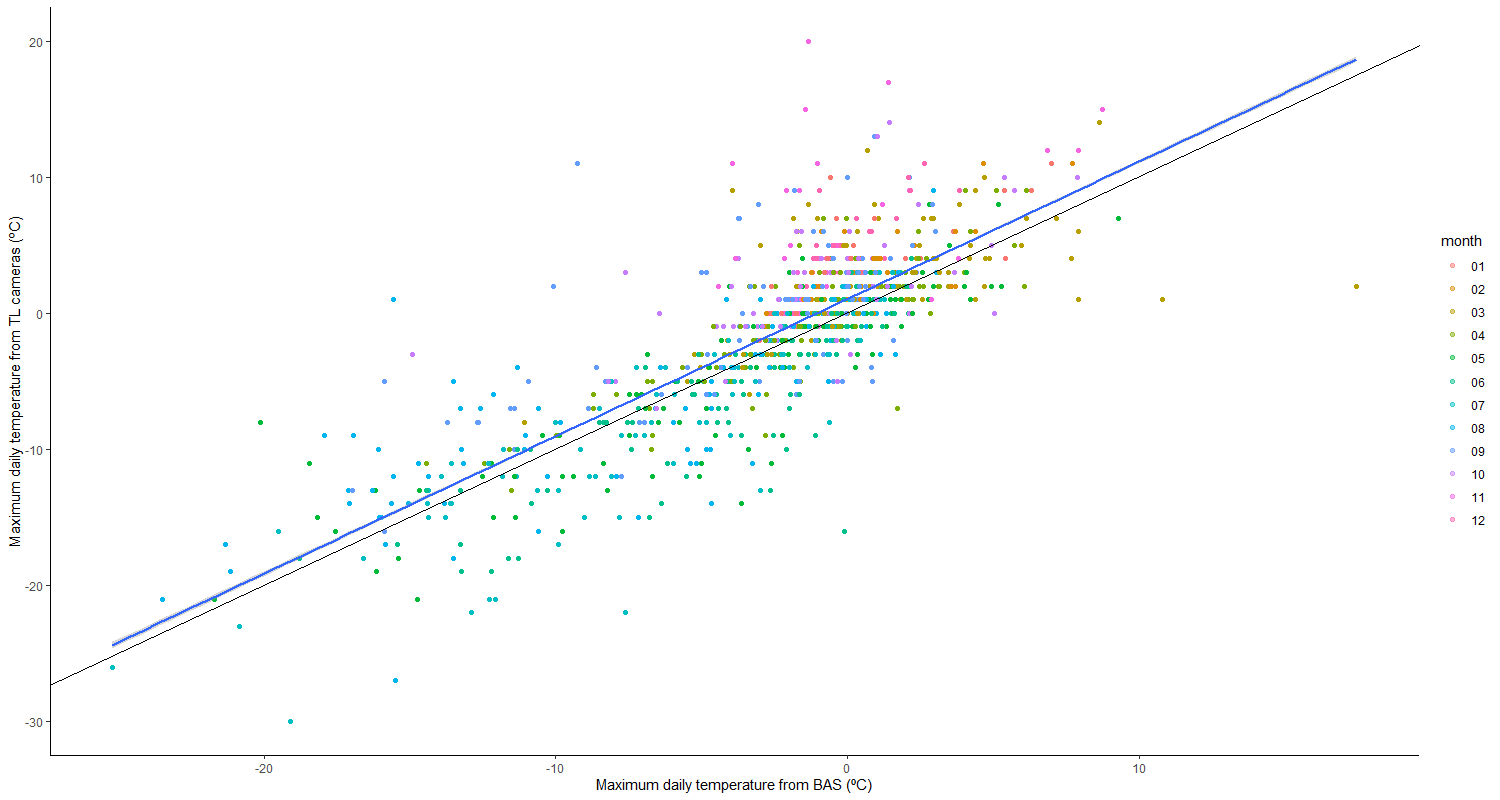


**Figure S2.3:** Daily maximum temperature values from our camera at Signy Island (SIGNa) are plotted against equivalent readings from the meteorological station at the British Antarctic Survey (BAS) station on Signy Island. Each datapoint is coloured according to the month they were measured in. A blue line showing the linear model fil is plotted in the foreground. A black line with 0 intercept and slope 1 representing an ideal equivalence between both datasets is plotted in the background for reference.

To test for accuracy, we used both the monthly mean and median temperatures (Figures S2.4 and 2.5) since they represent aggregated values for use in the phenology models. We calculated the average difference between measured TL values and those predicted by the model using the predict function in base R. The average deviation of the observed from the fitted values in the models are 1.427 for the median temperature and 1.455 for the mean temperature.

It is also worth noting that monthly means in warmer months are further biased as compared to the colder months (Figure S2.4). This is possibly due to the greater sensitivity of the mean to outliers compared to that of the median. We speculate the camera could have overheated on sunny days and contributed to increased temperature readings in warmer months. In any case median monthly averages are not consistently biased and therefore we will use these instead of the mean when conducting our analyses.


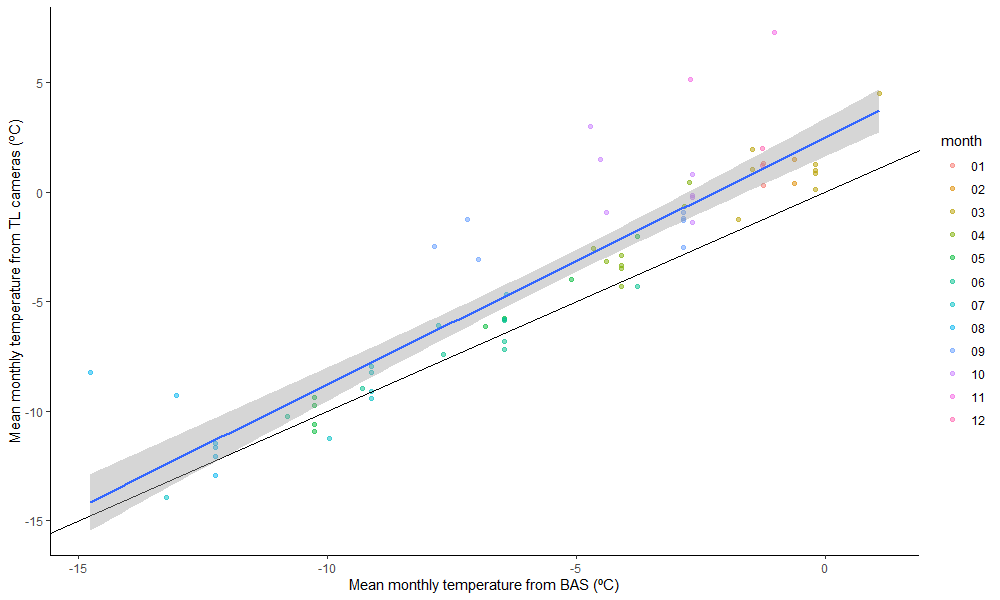
 **Figure S2.4:** Monthly mean temperature values from our camera at Signy Island (SIGNa) are plotted against the equivalent mean from the meteorological station at the British Antarctic Survey (BAS) station on Signy Island. Each datapoint is coloured according to the month they were measured in. A blue line showing the linear model fit is plotted in the foreground with its corresponding confidence interval. A black line with 0 intercept and slope 1 representing an ideal equivalence between both datasets is plotted in the background for reference.


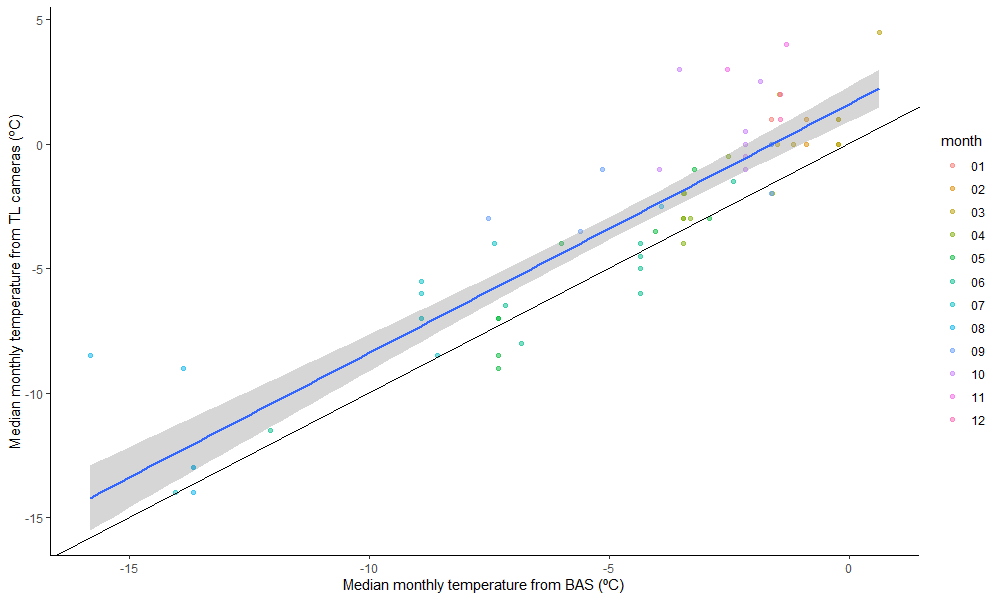


**Figure S2.5:** Monthly median temperature values from our camera at Signy Island (SIGNa) are plotted against the equivalent mean from the meteorological station at the British Antarctic Survey (BAS) station on Signy Island. Each datapoint is coloured according to the month they were measured in. A blue line showing the linear model fit is plotted in the foreground with its corresponding confidence interval. A black line with 0 intercept and slope 1 representing an ideal equivalence between both datasets is plotted in the background for reference.

In conclusion, our temperature measurements can be used, with two caveats. First, our daily readings are 1.6 to 1.8 degrees warmer depending on whether we use the median or mean (respectively). Second, the accuracy is limited and any changes under 1.4 degrees are within the measuring error of the camera.

**B) Net primary productivity**


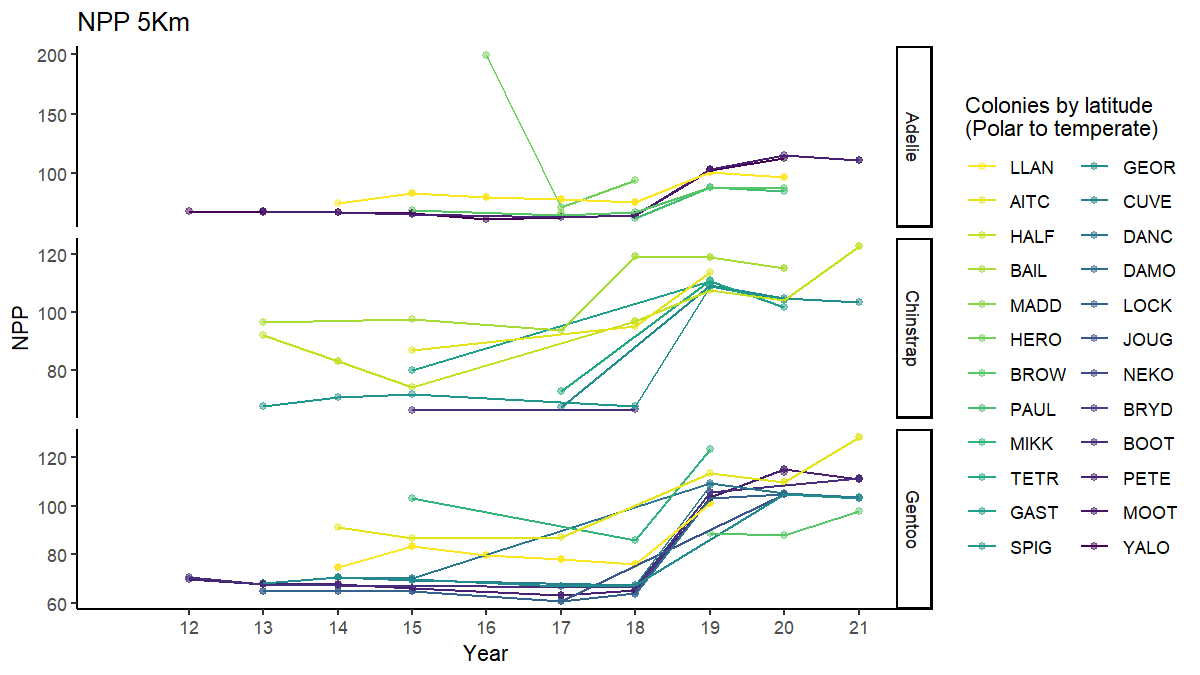


**Figure S2.6:** Changes in Net primary Productivity 5 Km around the study colonies in the Antarctic Peninsula over the study period.


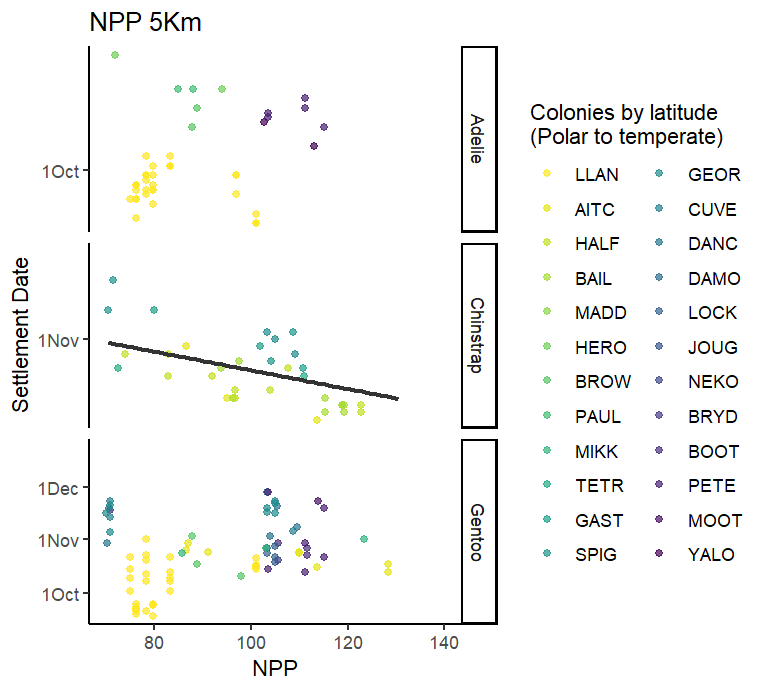


**Figure S2.7:** Relationship between Net Primary Productivity and Settlement data for all years and colonies of study available. Indicative regression line shown for chinstraps as it was the only species for which there was a significant relationship (See Table 1 in the main article).

**B) Sea Ice Cover**


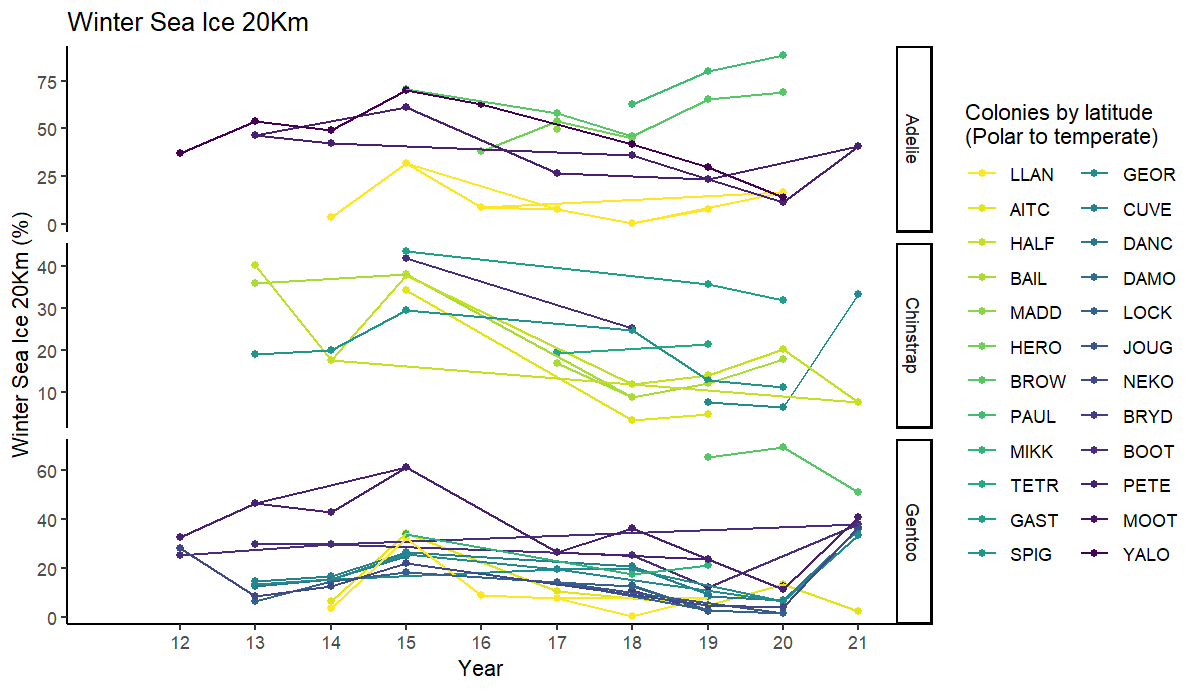


**Figure S2.8:** Changes in winter (Jul-Sep) Sea Ice Cover 20 Km around the study colonies in the Antarctic Peninsula over the study period.


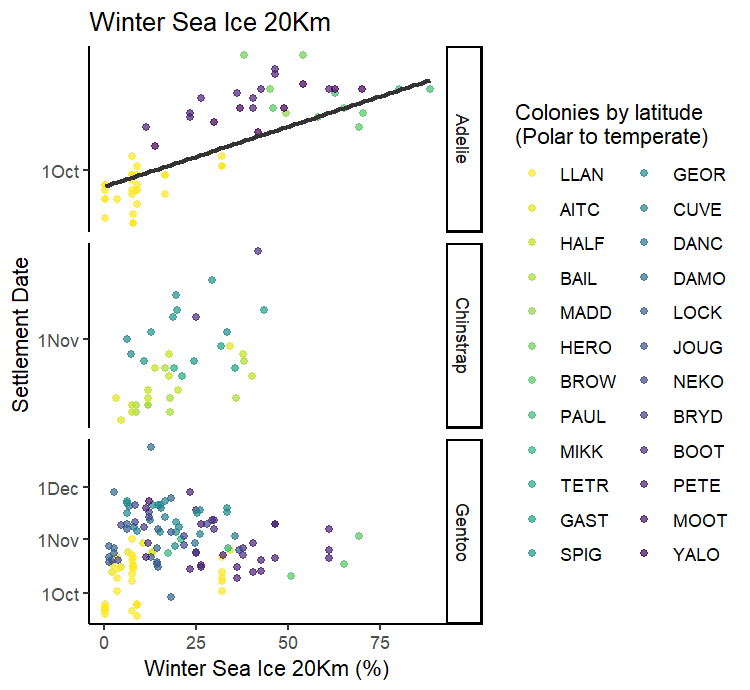


**Figure S2.9:** Relationship between winter Sea Ice cover and Settlement data for all years and colonies of study available. Indicative regression line shown for Adelies as it was the only species for which there was a significant relationship (See Table 1 in the main article).

**References**

Polar Data Centre, U. K, Convey, P., Geissler, P., Jobson, M., & Newsham, K. (2021). *Microclimate data from Jane Col, 2007-2016 [Version 2.0]*. NERC EDS UK Polar Data Centre. https://doi.org/10.5285/1BF0CFE7-A2E6-4C82-A0CD-D913876B6854
