# Supplementary material for: Record phenological responses to climate change in three sympatric penguin species
Source: J Anim Ecol. 2026 Jan 19;95(3):455–69. doi: 10.1111/1365-2656.70201 (PMC12957737; doi:10.1111/1365-2656.70201)
Supplement: Supplementary file 3 — Appendix S3: Model selection. Table S3.1. Mixed models used to calculate rate of change rate in settlement date for each species over the last decade (Negative slope meaning shift to earlier dates). Models were performed using the lmer function within the lme4 package in R. Settlement dates were tested against the continuous time variable year using colony. See Figure 2 in the main text for individual colony datapoints used in this model and their slopes. Table S3.2. Mixed models of monthly temperature increase over the last decade used to calculate warming rate in the study area. Models were performed using the lme function within the nlme package in R. Average midday temperature data for each month was tested against the continuous time variable year using colony as a random effect. See Figure 3 for individual colony slopes and SM Figure 2 for the monthly temperature datapoints used in these models. Table S3.3. Rates of advance per colony in settlement date for each species at each colony over the 2012‐2022 decade (Negative slope meaning shift to earlier dates). These were calculated using linear models (lm function within the lme4 package in R). Settlement dates were tested against the continuous time variable year. See Figure 2 in the main text for individual colony datapoints used in this model and their slopes. Figure S3.1. Monthly average estimates of direct temperature measurements from in‐built camera thermometers by year. Data from each colony is joined by a line and coloured according to the latitude of the colony (Darker meaning more southern/polar). [file JANE-95-455-s003.docx]

## Appendix S3: Model Selection

1. **Models for overall phenological shift**

**Table S3.1:** Mixed models used to calculate rate of change rate in settlement date for each species over the last decade (Negative slope meaning shift to earlier dates). Models were performed using the *lmer* function within the *lme4* package in R. Settlement dates were tested against the continuous time variable year using colony as a random effect. See Figure 2 in the main text for individual colony datapoints used in this model and their slopes.

| Mixed models:  **Species settlement date ~ year**.      Random Effect: **Colony** | | | |
| --- | --- | --- | --- |
| Month | Slope (days/year) | Std. Error | t-value |
| Adélie (AP) | -1.020 | 0.207 | -4.925 |
| Chinstrap (AP) | -1.042 | 0.158 | -6.578 |
| Gentoo (AP) | -1.296 | 0.446 | -2.692 |
| Gentoo (Scotia Sea) | -2.024 | 1.183 | -1.710 |

1. **Models of temperature increase in the region**

**Table S3.2:** Mixed models of monthly temperature increase over the last decade used to calculate warming rate in the study area. Models were performed using the *lme* function within the *nlme* package in R. Average midday temperature data for each month was tested against the continuous time variable year using colony as a random effect. See figure 3 for individual colony slopes and SM Figure 2 for the monthly temperature datapoints used in these models.

| Mixed models:  **Mean spring temperature ~ year**.      Random Effect: **Colony** | | | | |
| --- | --- | --- | --- | --- |
| Period | Slope (ºC/year) | Std. Error | t-value | p-value |
| August | 0.3764 | 0.05582 | 6.7428 | <2e-16 |
| September | 0.4095 | 0.0857 | 4.778345 | <2e-16 |
| October | 0.2038 | 0.05467 | 3.727369 | 3e-04 |
| November | 0.4474 | 0.04484 | 9.978066 | <2e-16 |
| Yearly | 0.3407 | 0.07249 | 4.700722 | <2e-16 |


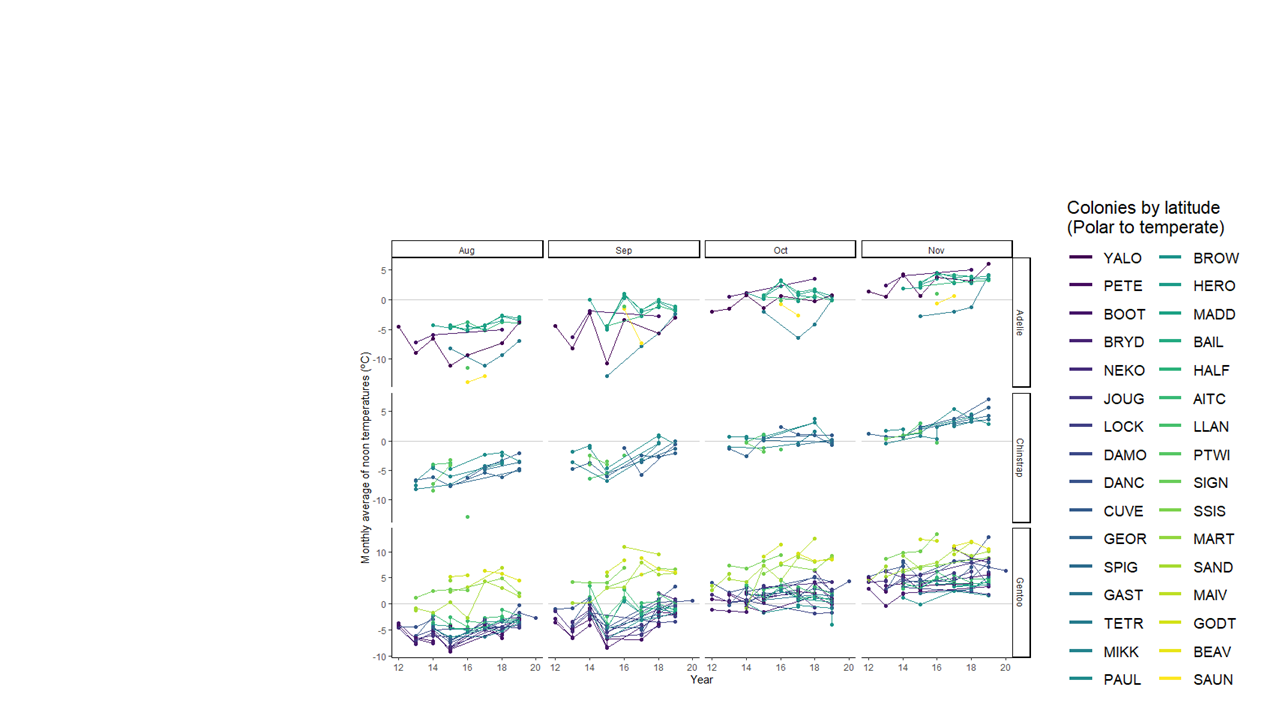
**Figure S3.1:** Monthly average estimates of direct temperature measurements from in-built camera thermometers by year. Data from each colony is joined by a line and coloured according to the latitude of the colony (Darker meaning more southern/polar).

1. **Rates of advance of the different colonies**

**Table S3.**3**:** Rates of advance per colony in settlement date for each species at each colony over the 2012-2022 decade (Negative slope meaning shift to earlier dates). These were calculated using linear models (*lm* function within the *lme4* package in R). Settlement dates were tested against the continuous time variable year. See Figure 2 in the main text for individual colony datapoints used in this model and their slopes.

| Colony | Species | Number of observations | p-value | Slope (rate of phenological advance) |
| --- | --- | --- | --- | --- |
| AITC | Gentoo | 8 | 0.108 | -1.299 |
| AITC | Chinstrap | 3 | 0.034 | -2.462 |
| BAIL | Chinstrap | 9 | 0.16 | -0.553 |
| BOOT | Gentoo | 9 | 0.001 | -2.608 |
| BROW | Adélie | 5 | 0.573 | -0.297 |
| BROW | Gentoo | 3 | 0.808 | -3.5 |
| COOP | Gentoo | 3 | 0.592 | 2.038 |
| CUVE | Gentoo | 7 | 0.077 | -2.399 |
| DANC | Gentoo | 6 | 0.845 | -0.259 |
| GAST | Chinstrap | 3 | 0.363 | -1.286 |
| GEOR | Gentoo | 12 | 0.611 | 0.458 |
| GEOR | Chinstrap | 4 | 0.355 | -1.286 |
| HALF | Chinstrap | 11 | 0.002 | -0.71 |
| HERO | Adélie | 3 | 0.333 | -3.5 |
| JOUG | Gentoo | 3 | 0.438 | -3.105 |
| LLAN | Adélie | 34 | 0.004 | -1.186 |
| LLAN | Gentoo | 32 | 0.968 | -0.056 |
| LOCK | Gentoo | 13 | 0.352 | -1.499 |
| MAIV | Gentoo | 5 | 0.099 | -4 |
| MART | Gentoo | 4 | 0.416 | 3.7 |
| MIKK | Gentoo | 3 | 0.754 | 0.731 |
| NEKO | Gentoo | 10 | 0.017 | -2.318 |
| PAUL | Adélie | 3 | 0.333 | 0.5 |
| PETE | Gentoo | 20 | 0.985 | 0.021 |
| PETE | Adélie | 11 | 0.002 | -0.99 |
| SAUN | Gentoo | 3 | 0.879 | -0.5 |
| SIGN | Adélie | 15 | 0.717 | 0.243 |
| SIGN | Chinstrap | 6 | 0.327 | -3.8 |
| SIGN | Gentoo | 3 | 0.437 | 9.5 |
| SPIG | Chinstrap | 6 | 0.099 | -1.084 |
| SSIS | Chinstrap | 6 | 0.079 | -1.705 |
| YALO | Adélie | 8 | 0.036 | -1.195 |

1. **Model building and selection for environmental variables related to phenological advance.**

In order to select the best fit we ran all models containing latitude plus several environmental variables. With so many variables we made a prior effort to eliminate highly collinear variables before the model-building stage. This is part of an effort to reduce the amount of variables included in the final model. Model comparison requires the analysis dataset to be exactly the same so including unnecessary variables might result in data exclusion if they contain an NA. This would result in an unnecessarily reduced dataset leading to less model power and singularity problems.

A) At-colony air temperature variables. Both extracted from the in-built camera temperature loggers. Their validation and use of the median over the mean are discussed in Appendix S2.

*Median October air temperature*, the month during which most settling occurs.

*Median spring air temperature*. Spring is considered September to November. This variable proved highly collinear in all three models and was therefore removed.

B) Proportion of freezing days. In order to monitor the possible loss of freezing days as a variable impacting phenology we built the two following variables:

*Days above zero in Spring.* This variable was discarded from all models due to its high collinearity. AP colonies had most of their September days at 100% freezing days and 0% for November, leaving October as the month providing the variability.

*Days above zero in October* was calculated as the proportion of days in which the temperature reaches above 1.8 degrees (due to camera bias See Appendix S2 and Table S2.1 inside) at least at one point during the day.

C) Sea-Ice Coverage.

*Mean sea ice concentration in winter* (Jul-Sep) using a 5km and a 20Km radius buffer.

*Mean sea ice concentration in October* using a 5km and a 20Km radius buffer.

In these variables we favoured the mean over the median as many days of zero ice would result in median-derived metrics with zero-inflated data. To avoid collinearity and row elimination due to NAs (model comparison requires the dataset to be exactly the same) only one of the four variables was used in model building. A prior analysis was conducted to understand which would be the most explanatory in each species on the basis of AIC.

D) Net Primary Productivity

Mean net primary productivity in October around the colony. Using a 5Km and a 20Km radius. Both were highly collinear so only the 5Km radius variable was considered in the end.

Models were built using only colonies at the Antarctic Peninsula since they are the only ones belonging to the latitudinal gradient (Figure 3 in main article). We computed the models using the lmer function of the lme4 package using camera as a random factor. Model comparison was performed using the *dredge* function in the *MuMIn* package in R. Final model selection was done on the basis of the smallest AIC. Conditional and marginal R^2^ estimates were obtained from the r.*squaredGLMM* function of the *MuMIn* package. Models were selected by means of their AIC. If two models were within 2 points of AIC were considered to be equally explanatory, in such case the model with the fewer explanatory variables was selected.

In selected models their assumptions (normality of residuals, normality of random effects, linear relationship, homogeneity of variance, multicollinearity) were tested for using the *check_model* function from the *performance* package. The resulting models for these three species are robust. All these models tested significantly better than their respective null models and all explain a sizable proportion of the variance as measured by the marginal and conditional R-squares of the three selected models.
