# Supplementary material for: Record phenological responses to climate change in three sympatric penguin species
Source: J Anim Ecol. 2026 Jan 19;95(3):455–69. doi: 10.1111/1365-2656.70201 (PMC12957737; doi:10.1111/1365-2656.70201)
Supplement: Supplementary file 4 — Appendix S4: Sub‐Antarctic colonies. Figure S4.1. Time series for the settlement date as estimated from Antarctic monitoring cameras for each species through the period of study. Settlement data for each camera is overlaid with their respective regression lines to ease interpretation and coloured according to their latitude from higher ‐more polar‐ latitudes (darker) to lower—more temperate—latitudes (brighter). Figure S4.3. Time series for the settlement date as estimated from Gentoo Sub‐Antarctic monitoring cameras through the period of study. Settlement estimates for the different colonies are coloured according to the suggested species segregation north and south of the polar front as suggested by various authors. Coloured Sub‐Antarctic estimates are overlaid on top of Antarctic settlement estimates for the corresponding species. Table S4.1. Model Selection for Scotia Sea Gentoos. Model selection was conducted including all variables of interest and dropping them off sequentially. Models are compared using AIC values although marginal and conditional R‐squared values are also shown to indicate the proportion of variance explained. This only includes Gentoo colonies north of latitude 60S. Table S4.2. Model Summary. The model that best explains arrival of Gentoo penguins at their respective Scotia Sea colonies corresponds to the model including latitude as the fixed effects while controlling for colony as the random effect. For more on model selection, see Appendix S3. [file JANE-95-455-s005.docx]

## Appendix S4: Sub Antarctic Colonies

Equivalent graphs to the Antarctic Peninsula plots in the main article. Figure S4.1 depicts the variation in settlement date across the years of study and figure S.4.2 shows their variation regarding median spring air temperature.


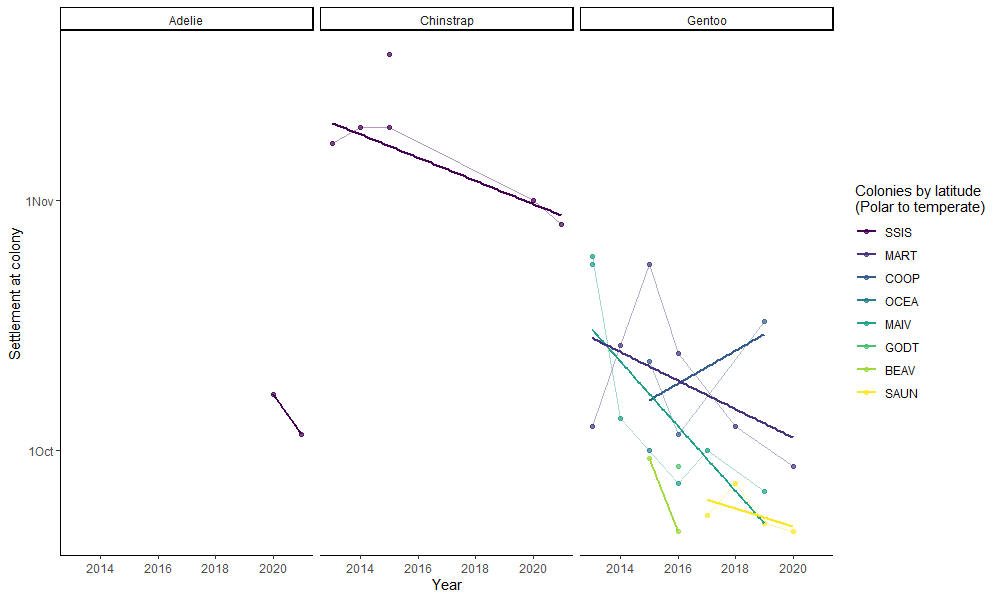


**Figure S4.1:** Time series for the settlement date as estimated from Antarctic monitoring cameras for each species through the period of study. Settlement data for each camera is overlaid with their respective regression lines to ease interpretation and coloured according to their latitude from higher -more polar- latitudes (darker) to lower -more temperate- latitudes (brighter).


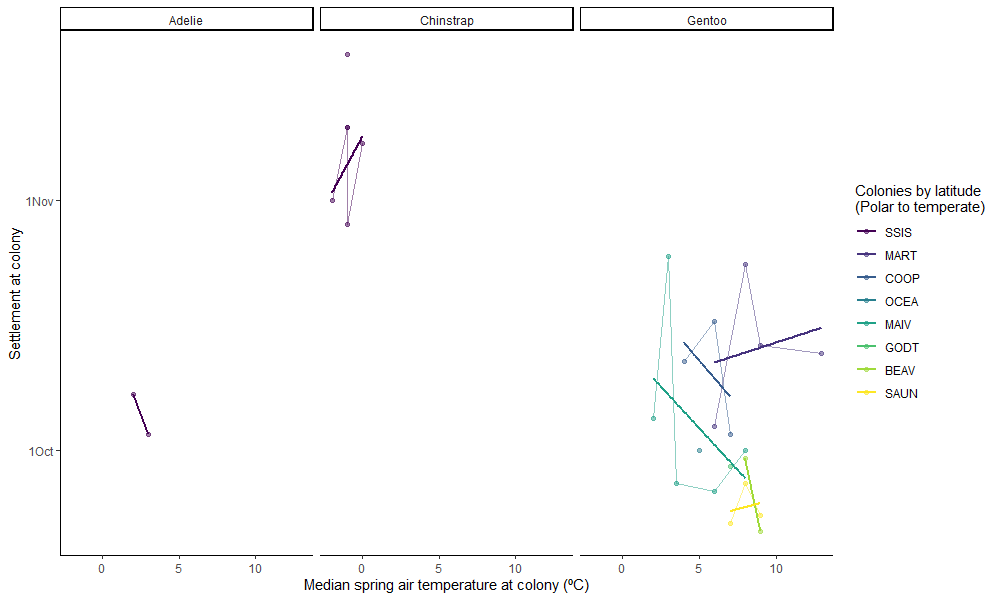
**Figure S.4.2:** Estimated settlement date by median spring air temperature for all cameras present at colonies around the Scotia Sea. Points belonging to the same camera are linked together and a linear regression is shown for each colony to ease interpretation. Regressions as well as linked datapoints are coloured by colony according to their latitude from more polar (darker) to more temperate (brighter).


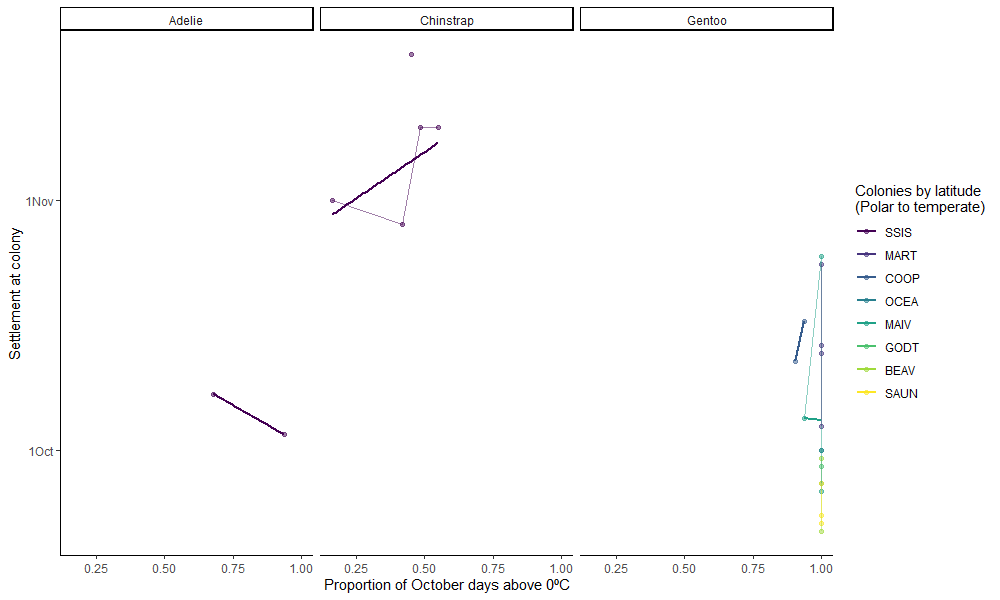


**Figure S.4.2:** Estimated settlement date by median spring air temperature for all cameras present at colonies around the Scotia Sea. Points belonging to the same camera are linked together and a linear regression is shown for each colony to ease interpretation. Regressions as well as linked datapoints are coloured by colony according to their latitude from more polar (darker) to more temperate (brighter).

**Differences within Gentoos**

There has been discussion about the possibility of separating gentoos into two species North and South of the Polar Front (Tyler et al., 2020). With our data, we cannot say that the Gentoos are phenologically distinct regarding settlement. All Gentoo Colonies in Sub Antarctic colonies show great overlap during settlement among themselves and with other Gentoo colonies in the AP (Figure S4.3)


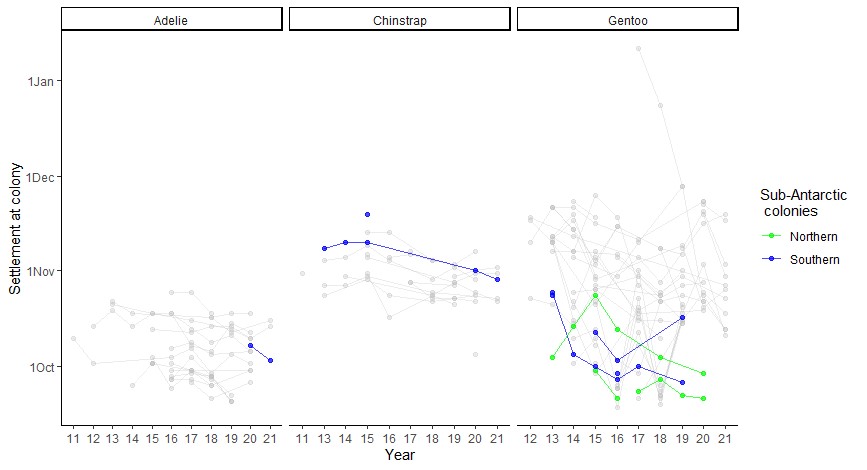
**Figure S4.3:** Time series for the settlement date as estimated from Gentoo Sub-Antarctic monitoring cameras through the period of study. Settlement estimates for the different colonies are coloured according to the suggested species segregation north and south of the polar front as suggested by various authors. Coloured Sub-Antarctic estimates are overlaid on top of Antarctic settlement estimates for the corresponding species.

In order to test for explanatory environmental variables behind the phenology shift we only conducted analysis on gentoos as they are the only species for which we have more than one colony. For Sub/Antarctic Gentoos we only conducted model selection including latitude and Spring median temperature, skipping days above zero as this is not a relevant metric for gentoos in the Sub Antarctic Islands (Figure S4.2). Finally, in order to avoid singularity effects (There are not enough datapoints for each colony to derive random effects from) we are going to group them by their island group (Patagonia, Falkland/Malvinas and South Georgia).

**Table S4.1: Model Selection for Scotia Sea Gentoos.** Model selection was conducted including all variables of interest and dropping them off sequentially. Models are compared using AIC values although marginal and conditional R squared values are also shown to indicate the proportion of variance explained. This only includes Gentoo colonies north of latitude 60S.

| Fixed effects | | Random effects | AIC | Marg.  Rsq | Cond Rsq |
| --- | --- | --- | --- | --- | --- |
| Median Spring Temp | Latitude | Colony group | 133.7802 | - | - |
| Median Spring Temp |  | Colony group | 139.4076 | - | - |
|  | **Latitude** | **Colony group** | **133.3258** | **0.423** | **0.474** |
| Null model (No fixed effects) | | Colony group | 139.6326 | - | - |

**Table S4.2 Model Summary.** The model that best explains arrival of Gentoo penguins at their respective Scotia Sea colonies corresponds to the model including latitude as the fixed effects while controlling for colony as the random effect. For more on model selection, see Appendix S3.

| **Scotia Sea**  **Gentoo settlement date model**  Fixed Effects: **Latitude**  Random Effects: **Colony** | | Variable (Random) | Variance explained (and %) |
| --- | --- | --- | --- |
|  |  | Island Group | 19.11 (30.1%) |
|  |  | Residual | 63.31 (69.9%) |
| Variable (Fixed) | Estimate | Std. Error | T-value |
| Intercept | -173.0 | 86.5 | -2.0 |
| Latitude | -5.020 | 1.612 | -3.114 |

**References:**

Tyler, J., Bonfitto, M. T., Clucas, G. V., Reddy, S., & Younger, J. L. (2020). Morphometric and genetic evidence for four species of gentoo penguin. *Ecology and Evolution*, *10*(24), 13836–13846. https://doi.org/10.1002/ece3.6973
