# Supplementary material for: Record phenological responses to climate change in three sympatric penguin species
Source: J Anim Ecol. 2026 Jan 19;95(3):455–69. doi: 10.1111/1365-2656.70201 (PMC12957737; doi:10.1111/1365-2656.70201)
Supplement: Supplementary file 5 — Appendix S5: Population trends. Figure S5.1. (Left) Total nest count for all Adélie colonies in this study present in the MAPPPD database. (Right) Same nest count data standardised to visualise trends. Figure S5.2. (Left) Total nest count for all Chinstrap colonies in this study present in the MAPPPD database. (Right) Same nest count data standardised to visualise trends. Figure S5.3. (Left) Total nest count for all Gentoo colonies in this study present in the MAPPPD database. (Right) Same nest count data standardised to visualise trends. [file JANE-95-455-s006.docx]

## Appendix S5: Population trends

Nest count for Adélie (Figure S5.1), Chinstrap (Figure S5.2) and Gentoo Penguin (Figure S5.3) colonies whose phenology is studied in the main paper. Data extracted from the MAPPPD database.


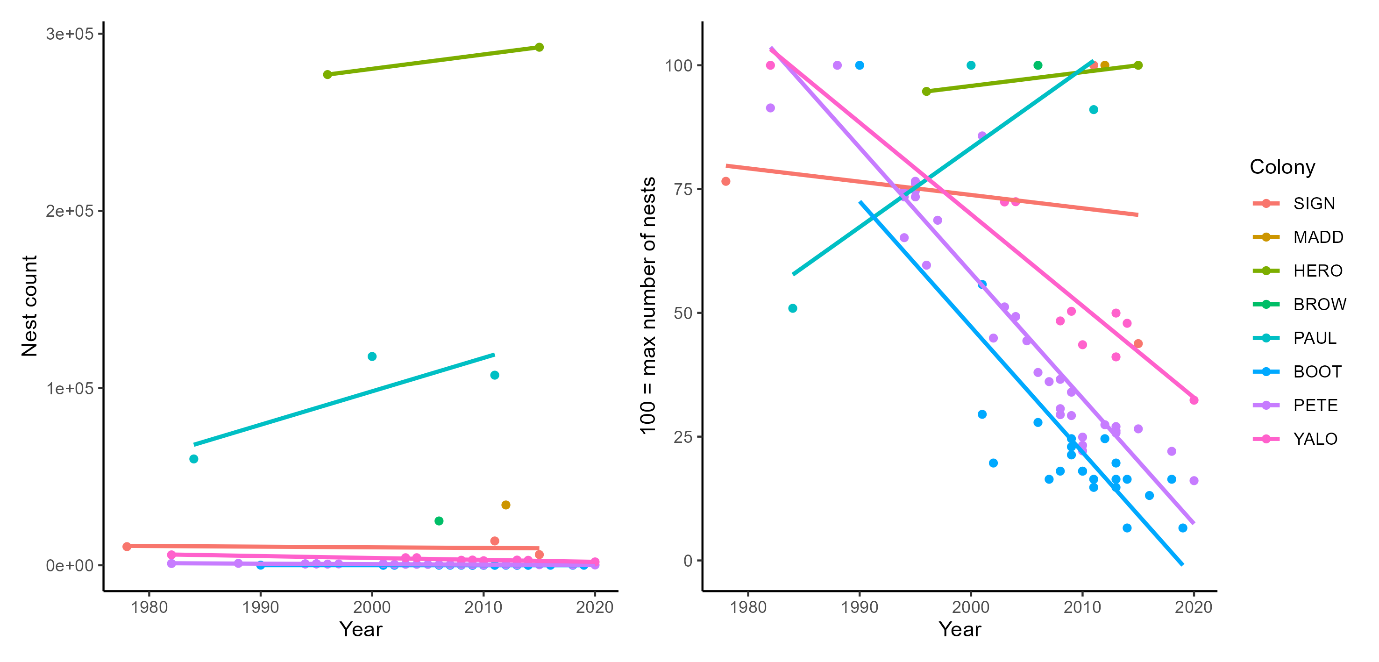


**Figure S5.1:** Left) Total nest count for all Adélie colonies in this study present in the MAPPPD database. Right) Same nest count data standardized to visualize trends.


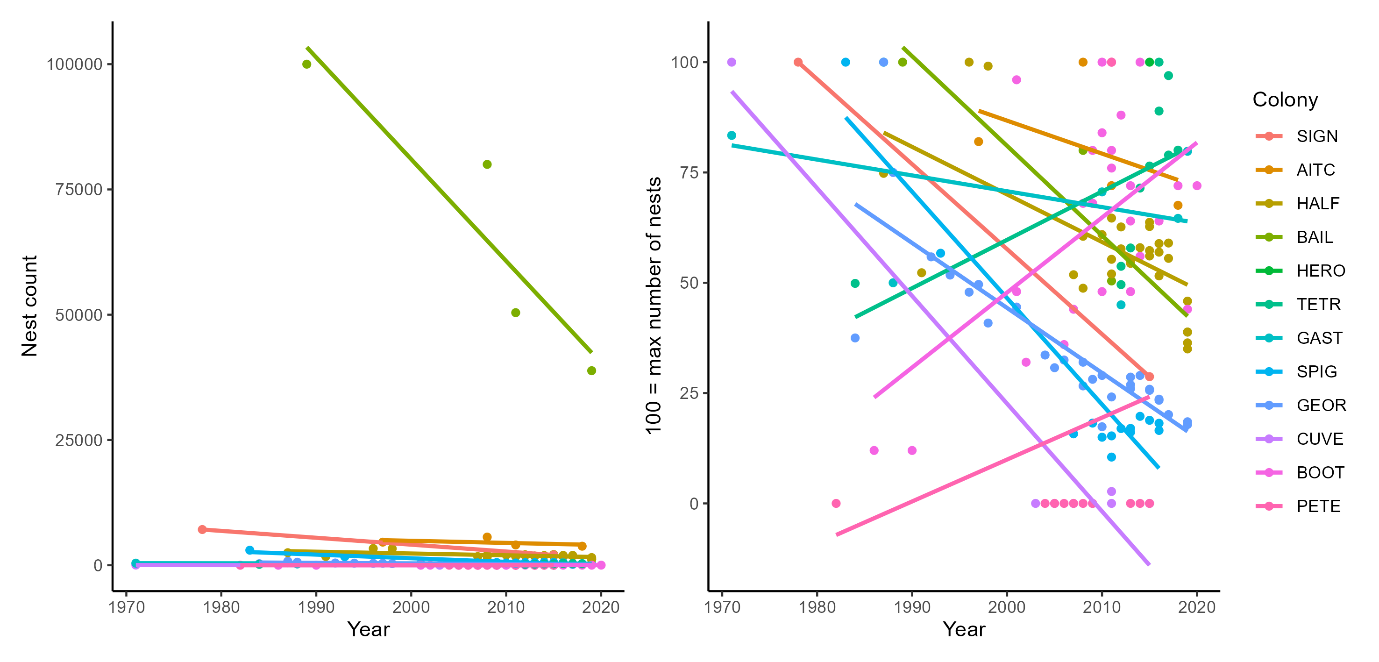


**Figure S5.2:** Left) Total nest count for all Chinstrap colonies in this study present in the MAPPPD database. Right) Same nest count data standardized to visualize trends.


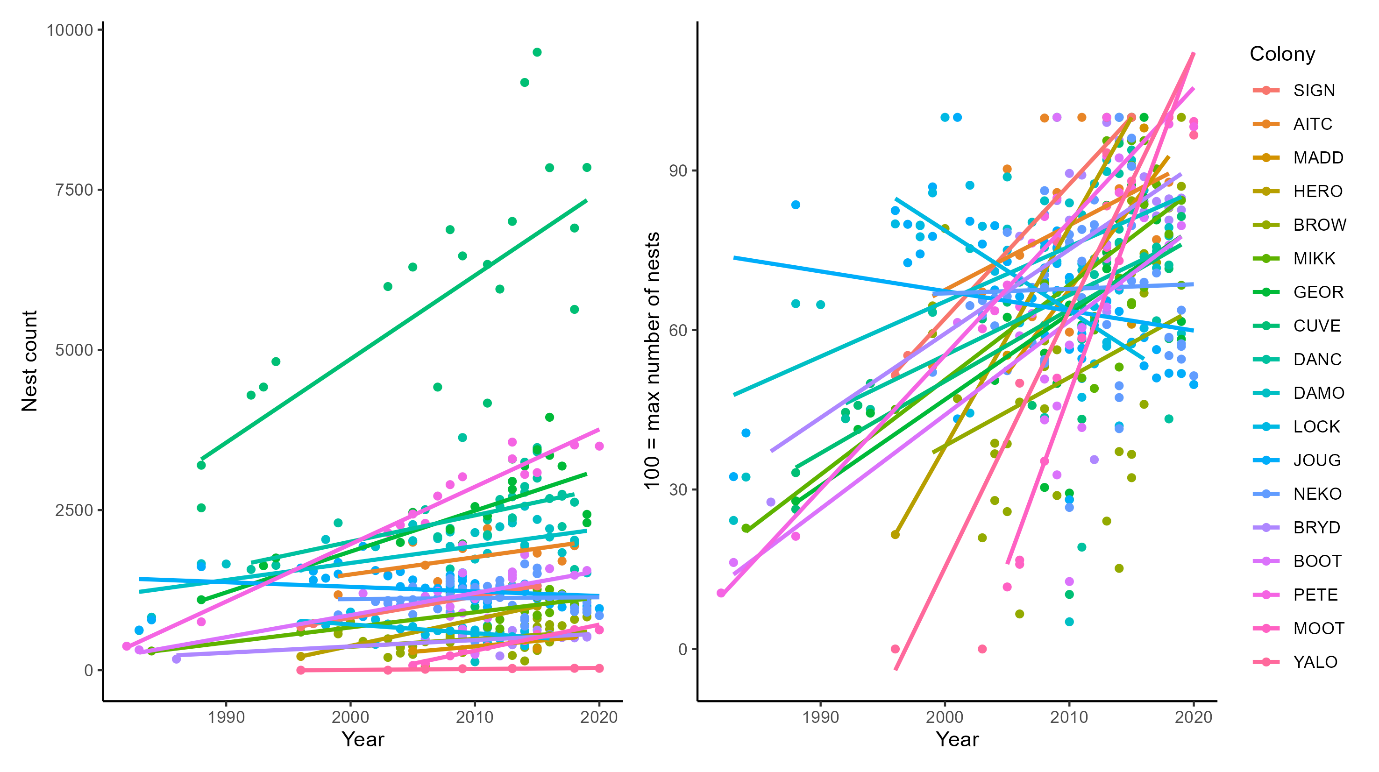


**Figure S5.3:** Left) Total nest count for all Gentoo colonies in this study present in the MAPPPD database. Right) Same nest count data standardized to visualize trends.
