# Supplementary material for: Record phenological responses to climate change in three sympatric penguin species
Source: J Anim Ecol. 2026 Jan 19;95(3):455–69. doi: 10.1111/1365-2656.70201 (PMC12957737; doi:10.1111/1365-2656.70201)
Supplement: Supplementary file 6 — Appendix S6. Settlement data compared to historical arrival data. [file JANE-95-455-s002.docx]

## Appendix S6: Settlement data compared to historical arrival data

This appendix contains a comparison of our settlement data to all historical data regarding “return to the colony” as reviewed by Black (2016). There are not many records of arrival data in the bibliography and many of them do not coincide with our colonies and species of study. This has left us with a handful of comparisons per species, enough for an indicative overview on how this data compares to historical observations. In order to keep two more comparisons we have compared historical data from Laurie Island to our cameras at Signy and historical data for Chinstraps at King George to our settlement data at the Aitcho Islands.

In all plots, settlement data used in this study is represented as dots joined by a line. Historical data is represented as tiles in the background covering the dates (y-axis) for which arrival has been described in the bibliography in that colony. The left-hand side of these tiles has been offset in order to allow better differentiation of periods and sources of data when there are several overlaid.

Western Antarctic Peninsula


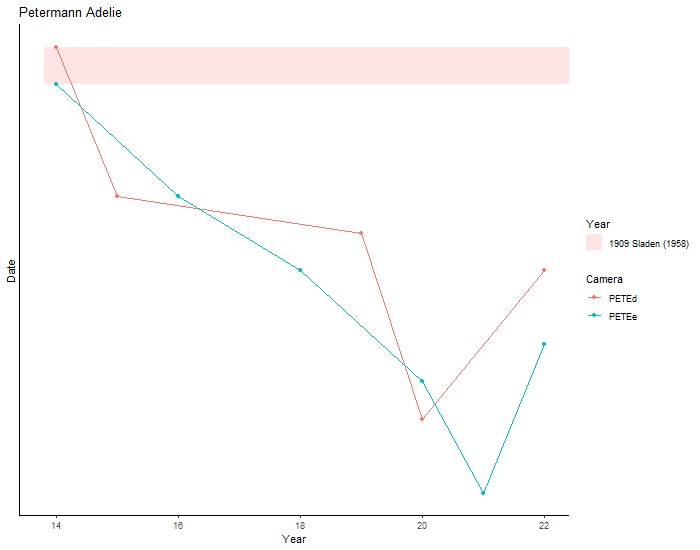


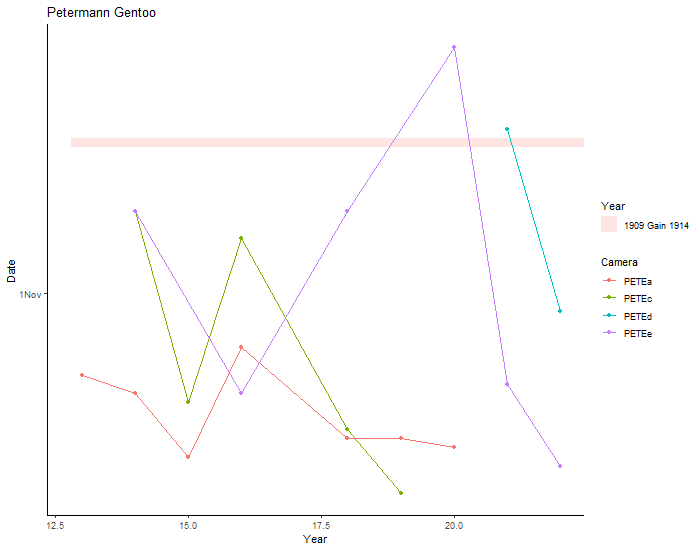


South Shetlands


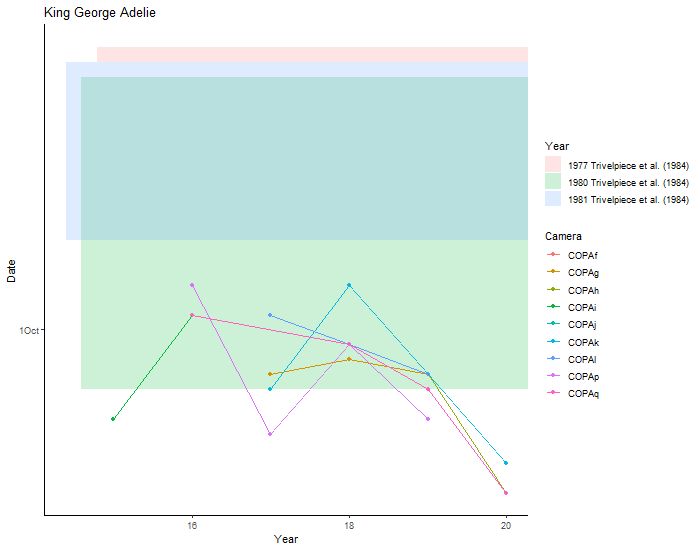


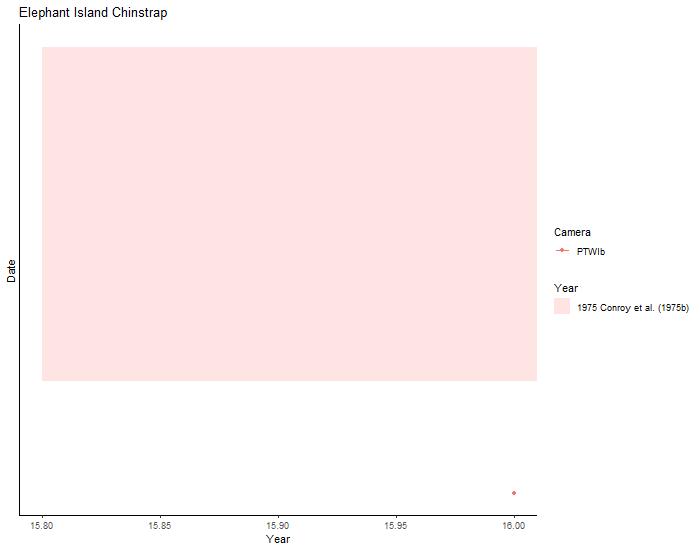


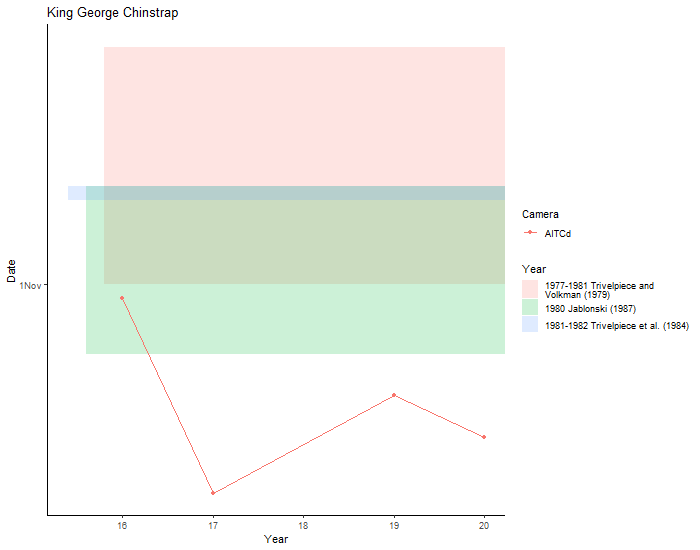


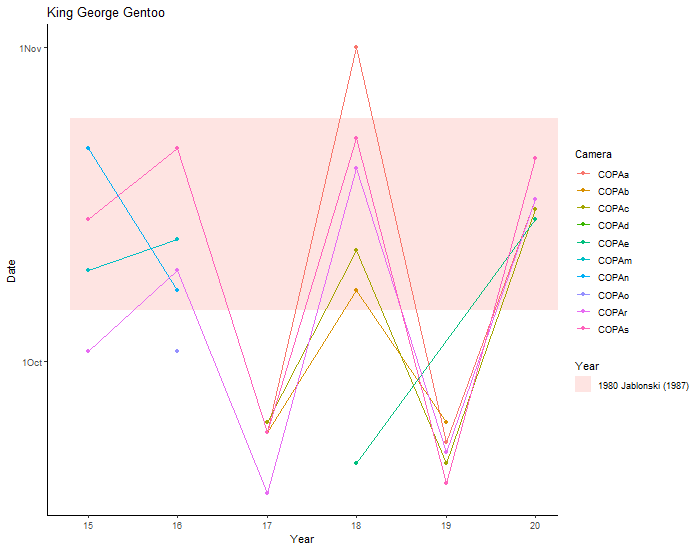


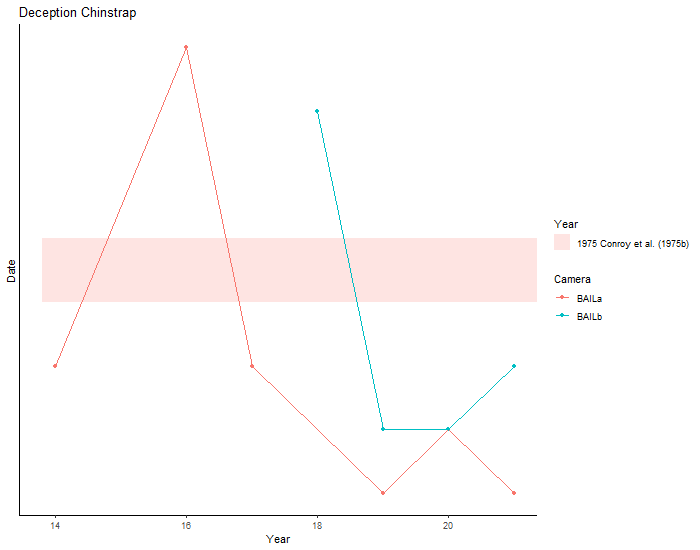


Weddell Sea


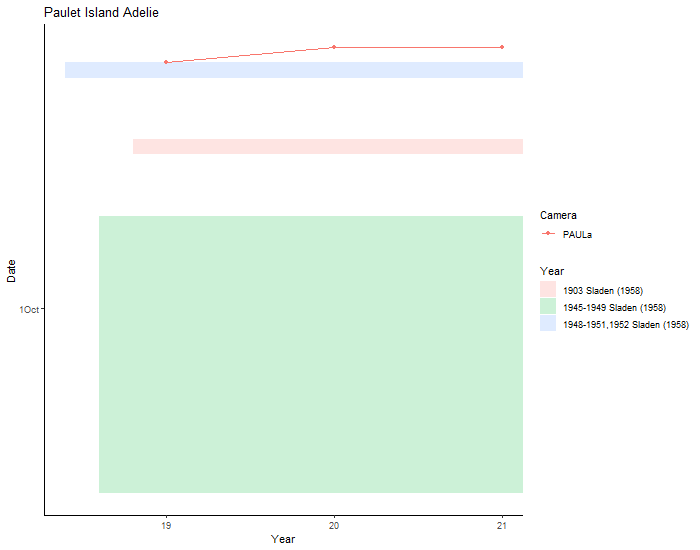


South Orkney


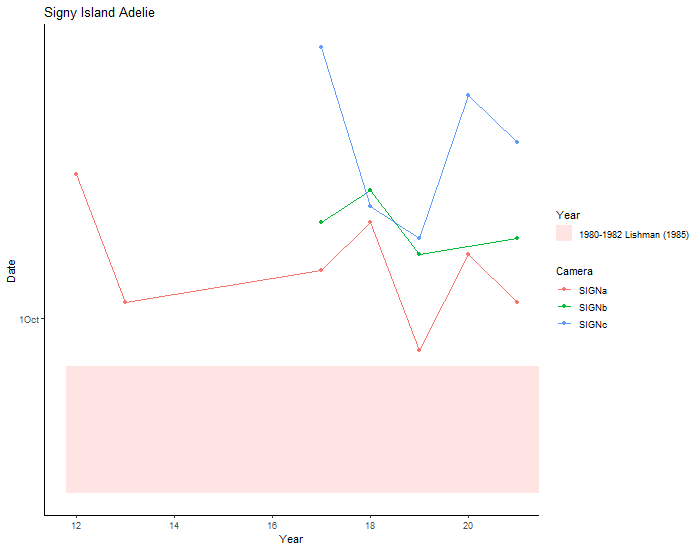


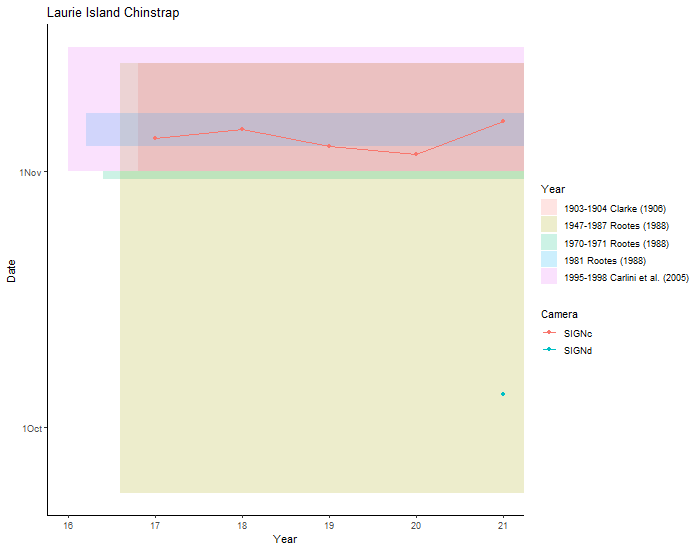


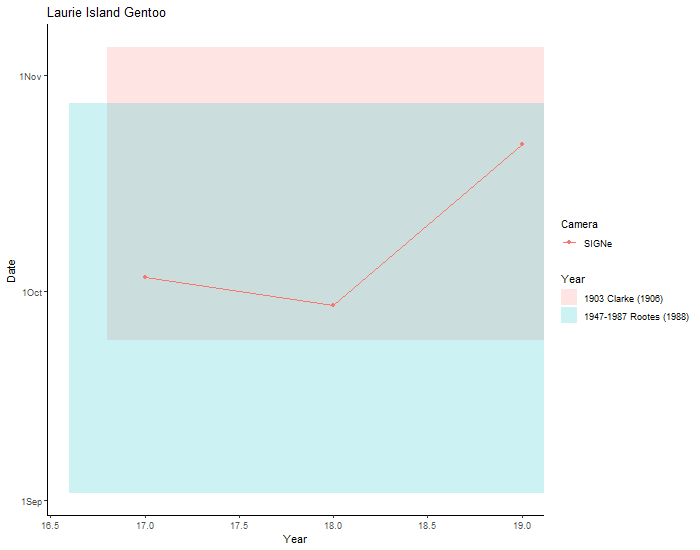


References:

Black, C. E. (2016). A comprehensive review of the phenology of Pygoscelis penguins. *Polar Biol*, 29.
